# A community-based physical activity intervention to prevent mobility-related disability for retired older people (REtirement in ACTion (REACT)): study protocol for a randomised controlled trial

**DOI:** 10.1186/s13063-018-2603-x

**Published:** 2018-04-17

**Authors:** Afroditi Stathi, Janet Withall, Colin J. Greaves, Janice L. Thompson, Gordon Taylor, Antonieta Medina-Lara, Colin Green, James Bilzon, Selena Gray, Heidi Johansen-Berg, Claire E. Sexton, Max J. Western, Jolanthe L. de Koning, Jessica C. Bollen, Sarah J. Moorlock, Naiara Demnitz, Poppy Seager, Jack M. Guralnik, W. Jack Rejeski, Ken R. Fox

**Affiliations:** 1 Department for Health, University of Bath, Claverton Down, Bath, BA2 7AY UK; 2 University of Exeter Medical School, St Luke’s Campus, Heavitree Road, Exeter, EX1 2LU UK; 3 School of Sport, Exercise and Rehabilitation Sciences, University of Birmingham, Edgbaston, Birmingham, B15 2TT UK; 4 Faculty of Health and Applied Sciences (HAS), University of the West of England (UWE Bristol), Frenchay Campus, Coldharbour Lane, Bristol, BS16 1QY UK; 5 Wellcome Centre for Integrative Neuroimaging, FMRIB, John Radcliffe Hospital, University of Oxford, Oxford, UK; 6 Wellcome Centre for Integrative Neuroimaging, Oxford Centre for Human Brain Activity, Department of Psychiatry, University of Oxford, Warneford Hospital, Oxford, OX3 7JX UK; 7 Department of Epidemiology and Public Health, University of Maryland, School of Medicine, 655 West Baltimore Street, Baltimore, MD 21201-1559 USA; 8 Department of Health and Exercise Science, Wake Forest University, Worrell Professional Center 2164B, PO Box 7868, Winston-Salem, NC 27109 USA; 9 Centre for Exercise, Nutrition and Health Sciences, School for Policy Studies, University of Bristol, Bristol, BS8 1TZ UK; 10 Global Brain Health Institute, Memory and Aging Center, Department of Neurology, University of California San Francisco, San Francisco, CA USA

**Keywords:** Physical activity, Disability prevention, Older adults, Randomised control trial, Mobility disability, Physical function, Frailty

## Abstract

**Background:**

The REtirement in ACTion (REACT) study is a multi-centre, pragmatic, two-arm, parallel-group randomised controlled trial (RCT) with an internal pilot phase. It aims to test the effectiveness and cost-effectiveness of a community, group-based physical activity intervention for reducing, or reversing, the progression of functional limitations in older people who are at high risk of mobility-related disability.

**Methods/design:**

A sample of 768 sedentary, community-dwelling, older people aged 65 years and over with functional limitations, but who are still ambulatory (scores between 4 and 9 out of 12 in the Short Physical Performance Battery test (SPPB)) will be randomised to receive either the REACT intervention, delivered over a period of 12 months by trained facilitators, or a minimal control intervention. The REACT study incorporates comprehensive process and economic evaluation and a nested sub-study which will test the hypothesis that the REACT intervention will slow the rate of brain atrophy and of decline in cognitive function assessed using magnetic resonance imaging (MRI). Outcome data will be collected at baseline, 6, 12 and 24 months for the main study, with MRI sub-study data collected at baseline, 6 and 12 months.

The primary outcome analysis (SPPB score at 24 months) will be undertaken blinded to group allocation. Primary comparative analyses will be on an intention-to-treat (ITT) basis with due emphasis placed on confidence intervals.

**Discussion:**

REACT represents the first large-scale, pragmatic, community-based trial in the UK to target the non-disabled but high-risk segment of the older population with an intervention to reduce mobility-related disability. A programme that can successfully engage this population in sufficient activity to improve strength, aerobic capacity, coordination and balance would have a major impact on sustaining health and independence.

REACT is also the first study of its kind to conduct a full economic and comprehensive process evaluation alongside the RCT. If effective and cost-effective, the REACT intervention has strong potential to be implemented widely in the UK and elsewhere.

**Trial registration:**

ISRCTN, ID:ISRCTN45627165. Retrospectively registered on 13 June 2016.

Trial sponsor: University of Bath. Protocol Version 1.5.

**Electronic supplementary material:**

The online version of this article (10.1186/s13063-018-2603-x) contains supplementary material, which is available to authorized users.

## Background

During old age, there is a population-wide transition from independence and adequate physical function towards frailty and an increased demand for health and social care support services. In particular, the prevalence of mobility-related disability increases rapidly with age [1]. Frailty and associated co-morbidities compromise quality of life for older adults and contribute major societal costs; directly to people who live with frailty, to friends and family providing care and losing productivity, and to health and social care services [2, 3].

Physical inactivity is one of the strongest predictors of mobility-related disability in older adults [4]. A fit and active older person has a 36% lower risk of developing functional limitations and a 38% lower risk of hip fracture [5]. In the UK-based Opal Plus cohort, older people who undertook at least an average of 25 min of moderate to vigorous physical activity (MVPA) per day at baseline received fewer prescriptions and were less likely to be admitted to hospital in an emergency 4 to 5 years later [6].

Clinical trials have provided robust evidence that physical features of frailty, such as reduced muscular strength or endurance, can be reversed, or at least the progression of these frailty indicators can be slowed, by undertaking an appropriate exercise programme [7, 8]. Unfortunately, older adults are the least active segment of the UK population. Less than 30% of 65–74-year-olds report any moderate-intensity physical activity lasting at least 10 min in the previous 4 weeks [9]. Health Survey for England (HSE) data indicate that among people aged 65 years and above, 36% of women and 27% of men reported a need for help in the last month with one or more activities of daily living (ADLs) such as getting up and down stairs, dressing, getting around indoors, or shopping [10]. These people are in transition from independence to frailty and have a great deal to gain if loss of function can be reversed and independence maintained.

Sedentary behaviour and mobility limitations in older people are more prevalent in socio-economically deprived sectors of the population [11]. Ethnically diverse groups in the USA have a significantly greater risk of a range of physical and mental health problems compared to their White counterparts, and subsequently suffer higher rates of morbidity and premature mortality [12]. Self-reported data from the HSE indicate that older (55 + years) Bangladeshi, Pakistani and Indian adults in England are less likely to meet physical activity guidelines compared to their White European counterparts [10]. Thus, exercise interventions that can successfully engage and retain sedentary and ethnically diverse groups of older adults could contribute to the reduction of health inequalities.

Lifestyle Interventions and Independence for Elders **(**LIFE) is a landmark study in the field of physical activity promotion in older adults [8]. LIFE was a multi-centre randomised controlled trial comparing the effects of a physical activity programme with a successful ageing educational programme in more than 1600 functionally impaired older persons, over an average follow-up of 2.6 years. The intervention reduced the risk of developing major mobility disability (defined as the inability to complete a 400-m walk test within 15 min) [8] by 18% relative to the control group (Hazard Ratio (HR) 0.82: 95% confidence interval (CI), 0.69–0.98). It also reduced the risk of persistent mobility disability by 28% (major mobility disability at consecutive time points) (HR 0.72; 95% CI, 0.57–0.91). The intervention group maintained an increase of 40 min/week (95% CI, 29 to 52; *p* < .001) in objectively assessed lifestyle-intensity activity (≥ 760 counts/min compared with the control group at 24 months of follow-up). These estimates are likely to be conservative as the study utilised an active control group which received a substantial health education/lifestyle intervention including weekly workshops for 6 months and monthly sessions for a further 18 months.

Building on the success of the LIFE study in the USA, the REtirement in ACTion (REACT) study aims to test the effectiveness and cost-effectiveness of a community-based physical activity intervention for reducing, or reversing, the progression of functional limitations in older people who are at high risk of mobility-related disability.

## Methods

### Trial design

The study protocol is presented in accordance with the Standard Protocol Items: Recommendations for Interventional Trials (SPIRIT) guidelines (Additional files 1 and 2).

The REACT study is a multi-centre, pragmatic, two-arm, parallel-group randomised controlled trial with an internal pilot phase and that incorporates comprehensive process and economic evaluations. Following identification and recruitment, 768 participants who meet the study inclusion criteria will be randomised to receive either the REACT intervention, delivered over a period of 12 months by trained facilitators or a minimal control intervention. The pilot phase will assess the feasibility of recruitment methods (allowing for some refinement if needed) and confirm adequate retention rates in the intervention and the study, prior to progressing with the main trial. A nested sub-study, led by the Wellcome Centre for Integrative Neuroimaging, University of Oxford, employing magnetic resonance imaging (MRI), will test the hypothesis that the REACT intervention will slow the rate of brain atrophy and of decline in cognitive function. Outcome data will be collected at baseline, 6, 12 and 24 months for the main study, with MRI sub-study data collected at baseline, 6 and 12 months. The REACT study was reviewed and approved by the National Health Service (NHS) South East Coast-Surrey Research Ethics Committee (15/LO/2082). The study is registered as a current randomised controlled trial (ISRCTN45627165).

The REACT study will be conducted at three UK sites – Bath/Bristol, Devon and Birmingham – allowing recruitment of a socio-economically and ethnically diverse sample including participants from urban, rural and semi-rural locations.

### Study population

The eligibility criteria are intended to identify 768 sedentary, community-dwelling, older people aged 65 years and over with functional limitations (i.e. who are at risk of major mobility limitations), but who are still ambulatory, i.e. they can still walk. This will be measured using a battery of objective physical function tests (Short Physical Performance Battery (SPPB)) to assess balance, walking speed and the ability to change from a sitting to a standing position. This generates a physical function score from 0 to 12 older adults with scores of 4–9 (inclusive) out of 12 will be eligible to take part in REACT. This is based on data showing that older adults with SPPB scores of 9 or less have substantially higher risk of major mobility disability 3 years later (odds ratio (OR) = 8.3 (95% CI, 3.3 to 20.67) compared with those with a score of 12 [8, 13]. Inclusion and exclusion criteria are summarised in Table 1.

**Table 1:** Eligibility criteria for participation in the REACT study and the magnetic resonance imaging (MRI) sub-study

Inclusion criteria	Aged 65 years or older and not in full-time employment Planning to reside in the target area (Bath/Bristol, Devon, Birmingham) for at least 24 months Score between 4 and 9 (inclusive) on the Short Physical Performance Battery (SPPB)
Exclusion criteria	Self-reported inability to walk across a room without a walker or the help of another person Existing major mobility limitation (defined as SPPB of 3 or less, or unable to complete the 4-m walk component of SPPB) Living in residential or nursing care Inability to attend the REACT physical activity sessions as scheduled A documented or patient-reported medical condition that would preclude participation, including: • Arthritis so severe it would prevent participation in physical activity • Parkinson’s disease or diagnosed dementia • Any terminal illness • Lung disease requiring use of orally administered corticosteroids or supplemental oxygen • Severe kidney disease requiring dialysis • Severe heart disease that would prevent participation in physical activity (e.g. chest pain when walking 100 or 200 yards or up a flight of stairs) • Implanted cardiac defibrillator • Cardiac arrest which required resuscitation • Severe uncontrolled psychiatric illness • Currently receiving radiation therapy or chemotherapy treatment for cancer • Awaiting knee or hip surgery • Major heart surgery (including valve replacement or bypass surgery) in the last 6 months • Unstable heart condition (e.g. uncontrolled arrhythmia, angina, heart failure or hypertension) • Spinal surgery in the last 6 months • Any other clinical condition that the person’s GP or clinician considers would make them unsuitable for participation in a physical activity rehabilitation programme to prevent decline of lower-limb functioning For the MRI sub-study: Contraindications for MRI scanning (assessed using CRIC Bristol Standard Operating Procedure for Screening Subjects for Safety to Scan (Ref MRI Local Rules and Procedures, 2011) History of neurological illness (e.g. stroke) Current treatment for a psychiatric illness Insufficient English to understand what participation entails and provide consent in English
Temporary exclusion criteria	Heart attack (or myocardial infarction), stroke, spinal surgery, hip fracture, hip or knee replacement within the previous 6 months Currently receiving physical therapy on legs or enrolled in another physical activity research or intervention study

### Recruitment

A range of recruitment strategies to identify suitable participants will be employed:

#### Primary care

General practitioner (GP) practices in the study catchment areas will be invited to participate through their local Clinical Research Network (CRN) and through existing networks. Where possible we will select practices to maximise diversity in terms of ethnicity, socio-economic status and (in Devon) rurality.

Practice staff will search the practices’ electronic patient databases for potentially eligible patients using the trial entry criteria (for criteria that are coded in the database). Lists generated from the searches will be further screened for suitability by a GP at each practice particularly to screen for items that are not included (or can only be partially covered) by the electronic searches. A Patient Approach Letter printed on the practice headed notepaper, a reply form and Participant Information Sheet (PIS) will be sent to suitable patients, with a reply paid envelope addressed to the research team at the local trial site. If the target recruitment rate is not achieved, practices will be asked to send out follow-up Participant Approach Letters to the same patients 14–21 days later. GPs and practice nurses may also offer the recruitment pack in surgery to patients who they consider would fit the REACT recruitment criteria.

#### Third-sector organisations

The principal investigators at each trial site will engage with third-sector and community-based organisations and authorities which work with adults over the age of 65 years. Professionals in these services will either approach potentially eligible service users and provide a brief summary of the study or invite the study researchers to give a presentation about the study, followed by provision of the Participant Approach Letter and PIS, if deemed appropriate. Publicity materials will be made available through libraries, supermarkets, post offices and GP surgeries.

#### Word-of-mouth and snowball sampling

To enhance recruitment, we will use word-of-mouth and snowball sampling techniques and employ the assistance of bi-lingual community champions. This approach will be particularly useful for engaging with ethnically diverse groups.

#### Local media

Recruitment will be supported by a low-cost public relations campaign targeting local newspapers, magazines, radio and community events.

Each REACT trial site will track recruitment methods to determine the most successful strategy for recruiting and for recruiting from diverse ethnic groups in particular.

### Eligibility screening

The eligibility of respondents will be assessed in a three-step sequential screening process:

1. *Initial self-selection*: the Participant Approach Letters and PIS will make it clear that we wish to recruit people who are still able to complete but have some difficulty with daily activities such as walking, climbing stairs and getting out of a chair
2. *Telephone-based screening*: after gaining verbal consent a preliminary telephone screen will check inclusion and exclusion criteria that can be assessed by telephone (e.g. self-reported inability to walk across a room), including a second check on medical exclusion criteria (e.g. recent heart surgery). Ability to attend intervention sessions will also be checked. Participants who do not meet the eligibility criteria will be thanked for their time and provided with an information pack including the Age UK/NHS guide to Health Ageing and sources of further advice and information
3. *Face-to-face screening sessions*: potentially eligible participants will then be invited to a group-based assessment session where they will have an opportunity to ask questions about the study and be asked to give written informed consent. The SPPB Gait Speed Test will be conducted first, and those who fail to complete the 4-m walk, or do not meet the other SPPB inclusion criteria, will be thanked for their time and provided with an information pack (as above). Participants who meet the eligibility criteria will be invited to complete the remainder of the baseline assessments
4. *MRI sub-study*: REACT participants who consent to discuss participation in the MRI sub-study will be screened for exclusion criteria using the CRIC Bristol SOP Screening Subjects for Safety to Scan [14]. This will be repeated immediately before the MRI scan

### Consent

Older adults who are willing to take part in REACT will be asked to provide written informed consent prior to commencement of the face-to-face screening sessions. The consent process will involve a brief recap of the PIS and an opportunity to ask questions prior to any of the face-to-face screening/baseline data collection processes commencing. Potential participants will be informed that they may, at any time, withdraw their consent to participate in the study without giving a reason, and without it affecting their relationship with their GP or the referring organisation and/or their future treatment and care.

### Randomisation/allocation

When 30 eligible participants have been recruited (enough to form one study group) they will be randomised to one of the two arms (physical activity (intervention) arm or social and education (control) arm using a secure, centralised, web-based randomisation website built by Peninsula Clinical Trials Unit (CTU). Randomisation will be performed using a minimisation algorithm to balance groups in terms of study site (Bath/Bristol, Birmingham, Exeter/Devon), age group, gender and initial functional ability (SPPB score). This algorithm has been built by the Peninsula CTU and uses the method proposed by Taves and extended by Pocock et al. [15, 16]. It maintains a stochastic element by computing probabilities proportional to the existing imbalance at the point of randomisation.

During the pilot phase, the randomisation will be 2:1 (intervention/control) in order to enable feasibility testing of intervention engagement and retention as early as possible. The main trial randomisation will be 1:1. Couples presenting together at the screening where both people are eligible and willing to be involved in the study will be randomised together to reduce contamination between study arms.

### Study intervention

The primary aim of the REACT study is to assess the effectiveness of a community-based physical activity intervention for reducing the progression of mobility-related functional limitations in older people who are at high risk of transition from independence to mobility-related disability.

#### Intervention arm

The intervention group will receive a standardised 12-month exercise programme designed for delivery in leisure/community centres where low-cost off-peak capacity is available. REACT will be delivered by qualified exercise professionals with experience of leading exercise classes in the community. We will collaborate with existing community-based organisations who have access to suitable venues and delivery staff. Sessions will be organised as group activities with up to 15 participants per group, but there will be individually tailored elements for aerobic exercise, strength work and exercise progression plans. Activities will include cardiovascular, strength, balance and flexibility exercises and daily lifestyle-based activity in the form of neighbourhood walking and active travel. Breaks in sedentary time will also be promoted. Social activities, such as post-exercise coffee meetings and community-based activities, will be organised to encourage a ‘social club’ atmosphere, promote long-term compliance and contribute to social well-being.

The intervention sets a long-term target for participants of 150 min of moderate-intensity activity per week, which is approached progressively. ‘Home-friendly’ exercises and written materials will be supplied to encourage participation in exercise in the home environment [17]. If participants miss two consecutive sessions, REACT leaders will telephone the participant to problem-solve ways for the participant to re-engage with the programme.

One novel element is the accompanying ‘REACT ambassadors’ scheme that provides the opportunity for participants to develop expertise and contribute as a local neighbourhood coordinator, or in other volunteering roles, determined by the provider. Our aim is to produce a pragmatic model of delivery that is rooted in the needs of the local community, that attracts a diverse population of older adults, is increasingly self-sustaining, and that has potential for application across the UK.

REACT will be delivered in two progressive phases (adoption and maintenance) and established behaviour-change techniques will be used to enhance motivation, to make realistic plans for sustainable activity, to pre-empt and overcome barriers, to engage social support and to use self-monitoring and self-regulatory techniques to support the maintenance of behaviour change. The REACT co-applicants will provide training in intervention delivery methods, including detailed session plans to ensure consistency in, and fidelity to, programme delivery.

*Start up* (adoption: weeks 1–8): the purpose of this phase is to stimulate initial increases in physical activity and fitness, to reduce any anxieties or concerns about exercise, and to build confidence and a sense of attachment to the programme. Each participant will receive a 45-min individualised, face-to-face introductory session which will be used to personalise the programme for starting levels and progression. Two 60-min physical activity sessions per week, plus 15–20 min of social time, will then be delivered by the REACT trainer.

*Build up* (adoption: weeks 9–24): a 45-min interactive educational/social session run by the REACT trainers will be added at the end of one of the two weekly sessions. These sessions will use evidence-based, person-centred behaviour-change strategies to build intrinsic motivation and self-efficacy. They will be designed to maximise enjoyment, social interaction and group identity [18]. Behavioural management will focus on self-regulation using goal setting, self-monitoring, reviewing of goals and problem-solving [19, 20]. A key focus will be on exploring and planning transition to more lifestyle-based activities. Pedometers will be introduced during these sessions to support the participant in the transition to the maintenance phase. After week 12, the exercise session frequency will be reduced to one per week but with an expectation that participants find an hour per week to exercise at home, in the neighbourhood or at a physical activity session in their local community. Participants will also be introduced to the REACT Ambassador training programme which will be delivered during the maintenance stage.

*Taking charge* (maintenance: weeks 25 to 52): the maintenance stage will focus further on home and neighbourhood-based activities while continuing with a weekly centre-based physical activity session followed by a short social session. Participants will enact action plans for physical activity outside of the REACT programme that were made during the transition phase and will be supported through group social/education meetings once a month. We will encourage groups to self-organise their own social interaction beyond the scope of the study and to consider doing activities together. Participants will be informed about local opportunities for physical activity in their community.

All intervention group participants will be offered the opportunity to be trained as *REACT Ambassadors* to help support the long-term sustainability of the programme. REACT Ambassadors will primarily focus on becoming local community activators facilitating local physical activity opportunities. This will help to increase maintenance activities and increase the frequency of meetings in the maintenance stage without adding to intervention costs.

Post intervention: REACT Ambassadors will help to sustain activities after the initial 12 months by organising group meetings and activities, and participants may be offered the weekly REACT sessions at a subsidised rate (subject to agreement with providers). The Ambassador programme is, therefore, designed to promote growth and increase sustainability.

#### Control arm

After completion of baseline assessments, participants allocated to the control group will be given information regarding healthy ageing. They will be invited to three 60–90-min group sessions over the 2 years of the study. These will consist of presentations and discussion groups on various aspects of healthy ageing (excluding physical activity) and incorporate socialising opportunities.

#### Intervention delivery

The REACT intervention sessions will be delivered at leisure or community centres provided by REACT collaborators during low-usage hours in economically and ethnically diverse areas in Bath, Bristol, Devon and Birmingham. REACT trainers will be qualified to at least Register of Exercise Professionals (REPS) Level 3 (Exercise Referral Diploma or equivalent) and will be experienced in delivering safe and effective exercise sessions to older adults. Social/education sessions may be delivered by other staff from the same organisation. All staff will attend a 2-day, standardised training workshop on the delivery of the REACT exercise and social/education sessions, based on a written programme of materials and manuals.

#### Intervention fidelity

We will include a range of the strategies outlined by the NIH Behaviour Change Consortium to reinforce intervention fidelity [21]. We will: (1) ensure ‘design fidelity’ by building our intervention around a clear logic model (Additional file 3: Figure S1); (2) recruit REACT trainers with appropriate skills and experience; (3) develop an accessible, standardised intervention manual; (4) implement the standardised REACT trainer training programme; (5) train more REACT trainers than needed to accommodate illness or withdrawal; (6) monitor delivery fidelity via recording of consultation meetings for a sample of three to four sessions per intervention provider and the application of a *fidelity checklist*: and (7) check for intervention ‘receipt’ and ‘enactment’ of appropriate levels of physical activity outside of the REACT sessions by including opportunities to check participant understanding of the correct performance of exercises and regularly review progress in the social-educational sessions. In addition, fidelity will be enhanced by incorporating a gradual transition to daily activity within the structure of the REACT intervention (i.e. withdrawal of one session per week after 12 weeks, reduction of social sessions to once/month after the first 6 months, with targeted planning of ongoing lifestyle-based physical activities around each transition).

#### Intervention theoretical framework

The REACT social and educational programme draws on two overlapping and mutually compatible theoretical perspectives, Social Cognitive Theory and Self Determination Theory. These theories provide the main principles and processes for supporting behaviour change [22, 23]. The Skills for Maintenance (SkiM) model has also been used to identify additional processes and techniques to promote maintenance of physical activity [24]. For further detail see the REACT logic model (Additional file 3: Figure S1).

### Assessments

A full list of measures and time points is presented in Table 2. The person conducting the assessments will check for completion of questionnaires before participants leave the assessment premises, and will make every effort to ensure that missed or spoiled questions are addressed.

**Table 2:** REACT schedule of assessment

Visit type	Scr1^1^	Scr2^2^	FU3^3^	FU4^4^	FU5^5^
Visit code		SV1	F06	F12	F24
Visit number		1	2	3	4
Telephone call	1				
Activity/assessment month	− 0.5	0	6	12	24
Form name					
Verbal consent	X				
Telephone screening (some elements of inclusion and exclusion criteria) (see Additional file 4)	X				
Written informed consent (see Additional file 5)		X			
Contact information update	X	X	X	X	X
Demographic, social, economic	X				
SPPB battery (see Additional file 6 for full CRF) [12]		X	X	X	X
Accelerometry		X	X	X	X
Height and weight		X		X	X
MoCA – Montreal Cognitive Assessment [13]		X	X	X	X
PASE questionnaire [14]		X	X	X	X
Dynometer (hand-grip strength)		X	X	X	X
Ageing Well profile (two sites full scale, one site social well-being scale only) [15]		X		X	X
Health-related quality of life (EQ-5D-5 L, SF-36) [16, 17]		X	X	X	X
Sleep Condition Indicator [18]		X		X	X
Pain (Western Ontario and McMaster Universities Osteoarthritis Index (WOMAC) [19]		X		X	X
Mobility Assessment Tool-short form (MAT-sf) [20]		X	X	X	X
Cognitive function (UK Biobank Healthy Minds Questionnaire) [21]		X	X	X	X
Medical history including medications, chronic diseases, cardiovascular disease and other conditions		X			
Falls Inventory including falls in last 6 months and injuries relating to falls		X	X	X	X
Health and Social Service Usage including use of primary, secondary or community care services in previous 6 months		X	X	X	X
*(MRI sub-study)* MRI scan, detailed cognitive assessment and gait analysis		X	X	X	
Process evaluation^6^			X	X	
Session attendance (intervention group only)			X	X	
Total contact time for each participant		X	X	X	
Physical activity related self-concept			X	X	X
Perceived tension of maintaining current PA			X	X	X
Perceived tension of maintaining current exercise		X	X	X	X
Autonomy in relation to PA		X	X	X	X
Competence for PA		X	X	X	X
Relatedness for PA		X	X	X	X
Enjoyment of PA		X	X	X	X
Perceived intrinsic benefits of PA (social, physical and emotional)					
Autonomy for strength-building exercise		X	X	X	X
Competence for strength-building exercise		X	X	X	X
Relatedness for strength-building exercise		X	X	X	X
Enjoyment of strength-building exercise		X	X	X	X
Perceived intrinsic benefits of strength-building exercise (social, physical and emotional)		X	X	X	X
Enjoyment of the REACT programme (intervention group)			X	X	
Credibility/identification with the session facilitators (intervention group only)			X	X	

^1^Telephone screening, ^2^Face-to-face screening (baseline assessment), ^3^6-month follow-up, ^4^12-month follow-up, ^5^24-month follow-up. ^6^Bespoke or adapted questionnaire developed for the REACT study. Full details of the process evaluation will be reported in detail elsewhere. The questionnaires are provided in Additional file 6

Abbreviations: *CRF* Case Report Form, *MRI* magnetic resonance imaging, *PA* physical activity, *SPPB* Short Physical Performance Battery test

### Sample size calculation

The primary aim is to assess the long-term (2 years) effect of a physical activity intervention on SPPB scores. A change of 0.5 points has been defined as a minimum meaningful change in SPPB score and 1 point is considered a substantial change [8, 25]. Based on data from the LIFE and LIFE-P studies, a difference between groups of 0.5 to 0.6 points in change in SPPB scores is feasible at 12 months and at 3 years. In the LIFE study, the standard deviation for the change in SPPB scores from baseline to 2 years was 2.2 and in the OPAL Plus UK-based study the standard deviation for change in SPPB over three years was 2.0 for participants with a baseline SPPB score of 8 or less. We have also assumed a two-sided significance level of 0.05 and that loss to follow-up will accumulate at 12.5% per year throughout the 2-year follow-up period of REACT.

To detect a between-groups difference of 0.5 points in SPPB change scores with a standard deviation of 2.0, with loss to follow-up at 12.5% per year, the required sample size is 384 per arm for 85% power using two sided 5% significance. The REACT study will therefore look to recruit a total sample of 768 participants. This sample size also provides 90% power to detect a difference in moderate intensity physical activity of 50 min per week (standard deviation (SD) 185 min/week) with significance at *p* = 0.05.

### Statistical analysis

Primary outcome analysis will be undertaken blinded to group allocation. Primary comparative analyses will be on an intention-to-treat (ITT) basis with due emphasis placed on confidence intervals. Using appropriate descriptive statistics, we will assess any imbalance between the trial arms at baseline and describe the characteristics of participants. The comparison of primary interest is the difference between the intervention and the control arm on SPPB score at the 2-year follow-up. This will be presented as between-group differences in means, 95% CIs and *p* values. Covariates in the model will comprise of baseline SPPB scores, centre, age group and gender.

Depending on the extent of missing primary outcome data, the primary analysis will be repeated using the complete data set generated using multiple imputations. Sensitivity analyses will be conducted to investigate the potential effects of missing data and of any co-interventions on the conclusions.

Secondary outcome analysis will be undertaken using the same general approach as for the primary analysis, using the baseline, 6-month, 1-year and 2-year follow-up data. This will include linear or logistic regression models for continuous or binary outcomes as appropriate.

#### Subgroup analyses

We will compare the primary outcome between sub-groups characterised by: (1) high vs. low adherence; (2) co-morbidity levels (one or less known chronic medical conditions vs. multiple co-morbidities); (3) socio-economic subgroups (using education, housing type and postcode), age categories (65–74 years and 75 + years); (4) gender and REACT site (Bath/Bristol, Devon, Birmingham) and (5) fallers and non-fallers (in 6 months prior to baseline).

#### Adjusted analysis

Using appropriate descriptive statistics, we will assess any imbalance between the trial arms at baseline and describe the characteristics of participants. As participants are randomised using the method of minimisation we are not expecting significant imbalance between groups. However, the minimisation variables (age, gender, SPPB) will be adjusted for in the statistical analysis.

### MRI imaging sub-study

#### Primary and secondary outcome data analysis

The primary analysis will be based on an intention-to-treat (ITT) principle. The primary outcome (change in adjusted hippocampal volume) will first be examined using a one-way analysis of variance (ANOVA) with group (exercise, control) as a between-subjects factor. An analysis of covariance (ANCOVA) model will then be carried out with the primary outcome as output, group as main factor, and age group, gender and education as covariates. If there is a main effect of group, single-sample *t* tests will be conducted to assess whether the rate of change in hippocampal volume differed from 0.

The secondary cognitive and behavioural outcomes will also be modelled using ANCOVAs. For the MRI secondary outcomes, specialised neuroimaging techniques, including non-parametric methods, will be employed. To examine the correlations between change in hippocampal volume and change in relational memory, Pearson’s correlations will be calculated.

#### Data presentation and analysis models

Continuous outcomes will be summarised by means and SDs (or medians and interquartile ranges (IQRs) if distributions are skewed). Categorical outcomes will be presented as numbers and percentages of patients in each category. Binary outcomes will be presented as numbers and percentages of patients in the category of interest.

#### Model assumptions

For all methods outlined, underlying assumptions will be checked using standard methods, e.g. residual plots. If assumptions are not valid then alternative methods of analysis will be sought. In particular, the underlying assumption of the linear impact of baseline covariates will be assessed and, if necessary, baseline covariates will be categorised and fitted as factors in the model. Age, gender and SPPB will be adjusted for in the statistical analysis.

#### Subgroup analyses

We will compare response to exercise based on SPPB score (ranges 4–7 and 8–9), adherence (high-low), age categories (65–74, 75 + years), gender and cognition (MoCA < 26 > 26, 26 + years).

### Economic evaluation

The economic evaluation will estimate the cost-effectiveness of the REACT intervention compared to control, for older people with major mobility limitations, from the perspective of the NHS and Social Care Services.

Using data collected within trial we will estimate the resource use and costs associated with delivery of the intervention, and the wider NHS-, social care- and participant-level resource use and costs over a 24-month follow-up. The primary component of resource use for delivery of the intervention is the time input (contact and non-contact time) for REACT trainers, and data will be collected on each activity by the trainer over the delivery of the intervention for each group, and across each phase of intervention delivery. Other areas of resource use (e.g. training, set-up, facility costs, cost of pedometers) will be recorded within trial by the trial coordinator. Data on use of health-, social care- and other participant-related resource use will be collected, using a participant self-report resource use questionnaire, at baseline, 6, 12 and 24 months. Data on resource use will be combined with national unit cost data obtained from available sources including Personal Social Services Research Unit (PSSRU) and NHS reference costs to estimate costs over the 24-month follow-up [26, 27]. The primary economic outcome measure will be the quality-adjusted life years (QALYs) derived from participant-reported EuroQoL five-dimension, five- level (EQ-5D-5 L) data, applying the area-under-the-curve approach [28], collected alongside the resource use questionnaire and using the UK tariff (presently recommended by NICE [29]). Data on estimated costs and QALYs by group will be presented, together with incremental QALYs and incremental costs, to present a policy-relevant cost-effective analysis (CEA).

Analysis will follow established methods for the conduct of economic evaluation in health technology assessment [30] and will be consistent with recommendations from NICE (UK) on ‘reference case’ cost-effectiveness analyses [31]. Findings will be reported in keeping with the CHEERS guidelines for cost-effectiveness studies [32]. CEA will be presented using base case estimates and uncertainty will be considered via detailed one-way and multi-way sensitivity and scenario analyses. The non-parametric nature of the distribution of resource use and cost data will be addressed using bootstrapping methods for estimation of confidence intervals around estimates of mean costs and incremental cost-effectiveness ratios (ICER). Results will include disaggregated data, as well as synthesis of costs and outcome data, and will include presentation of cost-effectiveness plane and cost-effectiveness acceptability curves to describe the likelihood of cost-effectiveness at different cost-effectiveness thresholds [33, 34].

To address missing data on costs and outcomes, multiple imputation will be used for making the assumption that the data are missing at random [35]. Given the longer-term nature of potential benefits from the REACT intervention, we will conduct evidence synthesis and decision-analytic modelling to assess the longer-term (lifelong) consequences of the intervention vs. control, including consequences in terms of health and social care costs.

### Process evaluation

The REACT process evaluation plan will follow the principles of the UK Medical Research Council guidance on process evaluation [36]*.* A full protocol for process evaluation is in development and will not be reported in detail here. However, the aims and methods are briefly summarised below*.* The purpose of the REACT process evaluation is to:

- Evaluate the feasibility of implementation, including barriers and facilitators, to inform future implementation and possible refinements of the intervention
- Evaluate the quality and quantity of intervention delivery to inform conclusions about intervention effectiveness
- Investigate the proposed mechanisms of change, outlined in the REACT logic model (Additional file 3: Figure S1) and seek alternative explanations if this model is not supported
- Understand the role of context to inform whether and how the findings can be generalised

Methods used in the process evaluation will include:

1. A mixed-methods assessment of intervention fidelity (quality of intervention delivery). This will include application of an intervention fidelity checklist to audio-recordings of a purposive sample of intervention sessions. This will generate a descriptive summary of delivery-quality scores. Each item will be scored using the Dreyfus competence-rating scale [37]. It will also generate a qualitative summary of the recorded sessions, highlighting examples of theorised and non-theorised intervention processes in practice, as well as examples of good and poor delivery. This analysis will help to interpret and contextualise the data on intervention adherence and effectiveness, providing explanations about why the intervention might work better for some people than others and what were the barriers to engagement with the intervention
2. A qualitative analysis of data to examine processes of change and elicit explanations for possible intervention success or failure. This will integrate data from: (1) semi-structured, longitudinal, individual interviews at 6, 12 and 24 months with a purposive sample of 24 participants in the intervention group (designed to sample diversity in terms of SPPB scores, session attendance, ethnicity, age, gender, service provider and area deprivation index) and (2) exit interviews at 24 months with a sample of participants and exercise leaders (after intervention completion). Topic guides will be piloted with the REACT service user advisory group prior to use. The analysis will capture individual narratives/within-person processes of change, as well as drawing out common themes. Emergent themes will be compared and contrasted with the theorised processes of change specified in the logic model. Processes of engagement with the intervention will also be explored. Using data collected at 12 and 24 months, factors influencing maintenance of physical activity/exercise will be assessed and linked to responses at 6 months. This will allow a qualitative description of participants’ experiences, potential pathways and barriers to maintenance. Techniques to enhance the objectivity and depth of the analysis may include cross-tabulation, negative case analysis and hypothesis testing, as well as respondent validation. Service users will also be involved in the interpretation of the data through workshops to discuss transcripts and the researchers’ interpretations of the data
3. Quantitative testing of hypotheses derived from the logic model. For example, we hypothesise that measures of theoretical determinants of behaviour change will change more in the intervention arm than in controls (between-group comparisons) and that these changes will be associated with between-group effects on physical activity (mediation analyses). Brief questionnaires assessing mechanisms of change suggested by Social Cognitive Theory and Self-Determination Theory (the main theoretical underpinnings of the intervention model) will be administered at baseline, 6, 12 and 24 months (see Table 2 and Additional file 6). We will also investigate the mediating and moderating effects of changes in physical activity on the primary outcome (physical function) and other outcomes of interest (cognitive function, sleep quality, well-being, depression) using multiple regression models

## Discussion

Breaking the spiral of decline that is characterised by loss of physical and cognitive function, reduced capacity to independently manage daily tasks and reductions in social interaction is fundamental to healthy ageing. It also has the potential to substantially reduce reliance on health and social care services as people grow older. This is particularly true for those who are at risk of mobility-related disability resulting from low levels of physical activity as they settle into changed routines after their primary working years. Strong clinical evidence supports the role of physical activity in reducing or even reversing this decline [8]. However, there have been few attempts to develop, and rigorously evaluate, feasible models of physical activity promotion for older people in community settings. In particular, there are no programmes that specifically target people at high risk of mobility-related disability, and few programmes are grounded in service-user and service-provider perspectives [38].

REACT represents the first large-scale, pragmatic, community-based trial in the UK to target the non-disabled but high-risk segment of the older population with an intervention to reduce mobility-related disability. This approach has many advantages. People in this category are still physically capable of engaging in a progressive exercise programme and have potential for prevention of further physical decline. A programme that can successfully engage them in sufficient activity to improve strength, aerobic capacity, coordination and balance would have a major impact on their prospects for sustained health and independence [8].

REACT is also the first study of its kind to conduct a full economic and comprehensive process evaluation alongside the RCT. These elements will test the effectiveness and cost-effectiveness of the REACT intervention, as well as build a deeper understanding of crucial mechanisms for (1) generating physiological and mental health benefit in this population and (2) promoting sustained change in physical activity. The MRI sub-study will provide significant advancements in the neuroscientific literature, adding comprehensive data on the impact of physical activity and functional ability on brain volume and cognitive function in later life.

If effective and cost-effective, the REACT intervention has strong potential to be implemented widely in the UK and elsewhere. The REACT study is likely to inform UK and international healthy ageing guidance and health promotion policies for the prevention of disability and the maintenance of independent living in older adults.

### Trial status

Enrolment into the study started in March 2016. Recruitment is expected to be completed by 31 October 2017 and final follow-up assessments by 30 November 2019.

## Additional files

Additional file 1: Completed SPIRIT (2013) Checklist detailing on which page of the protocol manuscript each of the relevant recommended items is addressed. (DOC 123 kb)

Additional file 2: Additional detail from the REACT Trial Protocol relating to the SPIRIT 2013 Checklist (Additional file 1). (DOC 117 kb)

Additional file 3: REACT process evaluation protocol and Logic model. (DOCX 128 kb)

Additional file 4: REACT Telephone Screening Form used to collect demographic data and undergo and initial eligibility assessment with potential participants. (DOCX 149 kb)

Additional file 5: REACT Participant Consent Form. (DOCX 135 kb)

Additional file 6: REACT Case Report Form including all measures and items used to collect outcome data as described in Table 2. (DOC 1427 kb)

## Abbreviations

AE: Adverse event
CEA: Cost-effective analysis
CI: Chief investigator
CRF: Case Report Form
CRN: Clinical Research Network
CTU: Clinical Trials Unit
DMEC: Data Monitoring and Ethics Committee
EC: European Commission
EU: European Union
EUCTD: European Clinical Trials Directive
EudraCT: European Clinical Trials Database
GCP: Good clinical practice
IB: Investigator Brochure
ICER: Incremental cost-effectiveness ratios
ICF: Informed Consent Form
IDMC: Independent Data Monitoring Committee
ISF: Investigator Site File
ISRCTN: International Standard Randomised Controlled Trials Number
ITT: Intention-to-treat
MRI: Magnetic resonance imaging
MVPA: Moderate to vigorous physical activity
NHS R&D: National Health Service Research and Development
PI: Principal investigator
PIC: Participant Identification Centre
PIS: Participant Information Sheet
QALY: Quality-adjusted life year
RCT: Randomised controlled trial
REC: Research Ethics Committee
SAE: Serious adverse event
SPIRIT: Standard Protocol Items: Recommendations for Interventional Trials guidelines
SPPB: Short Physical Performance Battery
TMF: Trial Master File
TMG: Trial Management Group
TSC: Trial Steering Committee
