# Supplementary material for: A community-based physical activity intervention to prevent mobility-related disability for retired older people (REtirement in ACTion (REACT)): study protocol for a randomised controlled trial
Source: Trials. 2018 Apr 17;19:228. doi: 10.1186/s13063-018-2603-x (PMC5905123; doi:10.1186/s13063-018-2603-x)
Supplement: Supplementary file 2 — Additional detail from the REACT Trial Protocol relating to the SPIRIT 2013 Checklist (Additional file 1). (DOC 117 kb) [file 13063_2018_2603_MOESM2_ESM.doc]

Additional file 7

Detail from the REACT Trial Protocol relating to the SPIRIT 2013 Checklist (Supplementary file 1)

| **Item no** | **Description** | **Detail from REACT protocol** |
| --- | --- | --- |
| 5b | Name and contact information for the trial sponsor | Name: Professor Jonathan Knight  Position: Pro-Vice- Chancellor for Research  Institution: University of Bath |
| 5c | Role of study sponsor and funders, if any, in study design; collection, management, analysis, and interpretation of data; writing of the report; and the decision to submit the report for publication, including whether they will have ultimate authority over any of these activities | No role of study sponsor and funders in study design, collection, management, analysis and interpretation of data, writing of the report and decision to submit the report for publication. NIHR, the funder, has the right to publish itself any non-confidential material generated from this project. NIHR will however consult with the PI if this is to occur. |
| 5d |  | **ROLE OF STUDY SPONSOR AND FUNDER**  The University of Bath will act as sponsor for the trial. The Chief Investigator and Trial Manager are employees of the University and will oversee the trial design, conduct, data analysis and interpretation, manuscript writing, and dissemination of results.  REACT is funded by the National Institute for Health Research - Public Health Research Programme. The funder expects the research team to conduct the study according to the trial as described and as set in the NHS ethics application. NIHR has the right to publish itself any non-confidential material generated from this project. NIHR will however consult with the PI if this is to occur.  **ROLES AND RESPONSIBILITIES OF TRIAL MANAGEMENT COMMITEES/GROUPS & INDIVIDUALS**  **Trial Management Committees**  **Trial Steering Committee**  The TSC will consist of an independent Chair with expertise in ageing and public health (Prof Yoav Ben-Shlomo, Bristol University); the CI; a service user representative, Dr Paul Bennett, an independent medical advisor, Professor Peter Thomas, an independent statistician from the University of Bournemouth; Professor Diane Crone of the University of Gloucestershire, an expert in the design, delivery and evaluation of health promoting interventions in primary care and in the community; Jameelah Ingram, a public health expert from Bath and North East Somerset Council and Dr Kate Walters, Director of the Centre for Ageing & Population Studies at UCL an expert in complex interventions in primary care and community settings. Representatives from the NIHR PHR programme will be invited to all TSC meetings and the trial statisticians, site PIs and health economist may be called on to attend as needed. The TSC will meet every 6-9 months from the start of the trial, providing overall supervision of the trial, monitoring trial progress and advising on scientific credibility. The TSC will consider and act, as appropriate, upon the recommendations of the Data Monitoring and Ethics Committee (DMEC) and will have responsibility for deciding whether the trial needs to be stopped on grounds of safety or efficacy. The TSC will be blinded to all information regarding treatment assignments until the database is locked for final analysis or if the DMEC recommends that results need to be reviewed.  **Data Monitoring and Ethics Committee**  A fully independent DMEC has been appointed which will report to the TSC. This will comprise of an independent chair Professor Dawn Skelton (Glasgow Caledonian University), Professor Paul Ewings, the Director of NIHR Research Design Service South West and Professor Kamlesh Khunti, Professor in Primary Care (University of Leicester). The CI, PIs and Chief trial statistician may be invited to attend to provide specific input by the DMEC Chair with the CI and statistician usually expected to attend the ‘open session’ section of the meetings. The DMEC will be responsible for the interests of the participants and its main role will be to make recommendations to the TSC as to whether the trial needs to be stopped for any ethical or safety reason (based on review of accumulating safety data). The DMEC will undertake safety data reviews every 12 months after recruitment begins, unless otherwise deemed necessary. This will include data on any adverse advents reported during the trial. Analysed data will be blinded, unless the DMEC identifies a specific need for unblinding.  **Trial Management Group**  The TMG will consist of the CI, all co-applicants, the trial manager, two people from our service user advisory group and the researchers at each trial centre. It will meet 4 times per year to ensure accurate implementation of the study protocol and the successful conduct and completion of the trial. The trial manager will also meet with the Chief Investigator and site leads for the three sites as needed, and each site will have its own site-specific meetings to discuss day to day project management issues. In accordance with the NIHR carbon reduction guidelines, organisation of teleconferences will be sought for two of the four TMG meetings and SKYPE or other online communications tools will be used to minimise environmental impact.  **PPI involvement**  REACT builds on several years of multidisciplinary work by this team aimed at understanding influences on the adoption and maintenance of physical activity in community-based activity programmes. Our Avon Network for the Promotion of Active Ageing in the Community (AVONet) (MRC Lifelong Health and Wellbeing – Collaborative Development Network (Ref 90543)) used focus groups and workshops with service providers, older people, international experts and service commissioners to assess the needs of older people and their communities for physical activity promotion. The REACT study was considered by our AVONet service user, service provider and commissioner stakeholders to be suitable for delivery across a range of socio-economic and cultural populations. The REACT protocol has been developed based on this input. The Trial Management Group was closely involved in the development of the study protocol and three people from our service user advisory group (research partners) form part of that committee. The Trial Steering Committee which approved the protocol prior to submission included a service user representative. In addition the draft protocol was open to consultation by our service user representatives, our public health expert and members of community organisations. |
| 7 | Specific objectives or hypotheses | **REACT primary hypothesis**  Compared with the control group, participants allocated to receive the REACT programme will have significantly reduced mobility-related limitations, as indicated by SPPB score, at 24 months of follow-up.  **REACT secondary hypothesis**  Compared with the control group, participants allocated to the REACT programme will significantly increase their levels of moderate intensity physical activity, health-related quality of life, cognitive function, ability to perform the activities of daily living, mental and social well-being and have reduced pain and improved sleep quality at 24 months. |
| 11b | Criteria for discontinuing or modifying allocated interventions for a given trial participant | The Trial Steering Committee and the Data Monitoring and Ethics Committee will oversee all patient safety issues, which the REACT independent medical advisor, Dr Paul Bennett, will review in detail.  The University of Bath standard operating procedure for reporting research related Adverse Events (AEs) will be adopted. The detailed process for the reporting of Adverse Events and Reactions is outlined in the REACT study protocol and the IRAS submission. The DMEC will monitor and analyse data on any adverse events reported during the trial. |
| 12 | Primary, secondary, and other outcomes, including the specific measurement variable | **Primary Outcomes**  The Short Physical Performance Battery (SPPB) score at 24 months. SPPB is an objective battery of functional performance tests (observed ability to complete a repeated sit-to-stand task, a standing balance test and a gait speed assessment). The resulting score ranges from 0 to 12. The SPPB can usually be completed in 5 minutes with the use of a stopwatch, a 4-m tape and a chair. Inter-rater reliability is reported as 0.9 and test–retest reliability is 0.72. The SPPB has been shown to predict both mobility-related disability (inability to complete a 400m walk in 15 minutes) and ADL disability (using Barthel Index ADL scores). SPPB score provides a reliable estimate of future risk of hospitalisation and decline in health and function in older adults. Risk of mobility-related disability over a three-year period shows a strong graded response across the range of SPPB scores (OR = 26.9; 7.7; 8.3; 3.4 for SPPB <= 7; SPPB <=8, and SPPB <= 9; SPPB ,=10, respectively. Based on these associations and other data, a 0.5 difference (effect size 0.25) is considered to be a clinically meaningful change in SPPB score.  **Secondary endpoints/outcomes**  1. Change in minutes of moderate intensity physical activity, as measured by accelerometer data using a protocol successfully used in previous studies. We will use wrist-worn accelerometers as they provide high compliance rates, minimal burden to participants, and they are waterproof minimising the risk for participants to forget to put them back on after swimming or having a shower (common problems with waist worn accelerometers).  2. Sedentary time and breaks in sedentary time per day assessed by accelerometry.  3. Self-reported physical activity (PASE questionnaire). 4. Hand grip strength of the dominant hand using a digital dynamometer (predictive of functional limitation)  5. Brief measures of mental well-being the Social Well-Being scale of the Ageing Well Profile (6 items)); Sleep Condition Indicator (8 item) pain (Western Ontario and McMaster Universities Osteoarthritis Index (WOMAC) (5 item).  6. Health-related quality of life (EQ-5D, SF-36).  7. Activities of daily living (ADL) will be measured with the Mobility assessment tool-short form (MAT-sf (See Appendix 5)), SF36 and EQ-5D.  8. Medical history, Falls Inventory and Health and Social Service Usage  9. Cognitive function will be measured using the UK Biobank Healthy Minds Questionnaire which assesses memory, attention and executive function  10. Cognitive function – *(fMRI imaging substudy).* Our colleagues at Oxford University will test the hypothesis that a physical exercise intervention slows the rate of brain atrophy and of decline in cognitive function. Measures include a brief battery of paper and pencil and computerised tests to assess memory, attention and executive function; structural and functional brain MRI measures and gait analysis for a sub-sample of participants.  11. The cost-effectiveness analysis will present results against the primary outcome measure, and against cost per QALY, using a generic preference-based health status measure (EQ-5D) for base case estimates and uncertainty will be considered via detailed sensitivity analyses using the (SF-36). |
| 16c | Who will generate the allocation sequence, who will enrol participants, and who will assign participants to interventions | Confirmation that randomisation has been performed will be communicated in a blinded fashion to investigator site staff and key members of the central research team. Communication will be achieved via emails automatically generated by the randomisation website.  The CTU will send the study ID numbers of intervention and control participants to a departmental administrator at the University of Bath. The administrator, who will have no involvement in the research elements of the study, will telephone participants to inform them of their allocation and send them a confirmation letter using the contact details collected at the baseline clinic visit.  During the telephone call to the control group, participants will be invited to their first social/education group session which will be held 10-12 weeks after allocation. They will also be mailed a REACT information pack containing healthy ageing advice. A thank you for participating card will be sent to confirm the date of the social/education group session. Letters to participants in the intervention group will advise participants of the date, venue and transport arrangements for their attendance at REACT sessions. The departmental administrator will follow this up with a telephone call shortly before the day of the first session to re-confirm the arrangements and discuss any practical issues. |
| 17a | Who will be blinded after assignment to interventions (eg, trial participants, care providers, outcome assessors, data analysts), and how | It is not possible to blind study participants to treatment allocation in behavioural intervention studies and this is not a problem in pragmatic trial designs, which aim to estimate the benefits of the intervention over and above usual or standardised care^50^. However, we will take steps to ensure that data collectors, statisticians and the research team remain blinded to group allocation. At follow-up data collection visits, patients will be asked not to reveal which group they are in. Allocation codes will be locked away by the CI until the database is closed for analysis.  Data will be coded so that those performing the statistical and economic analyses will also be blinded. Given the study design, we do not anticipate a substantial risk of contamination (i.e. exposure of the control participants to the REACT intervention). However, as part of their briefing on entry to the study (and at follow up measurement visits), participants in the intervention arm will be asked not to share or discuss the content of the intervention sessions with any control participants they may be in touch with, for the duration of the study. The possibility of contamination of control patients by intervention deliverers will be minimised by giving clear instructions to the intervention deliverers not to provide intervention materials or information to any participants not assigned to the intervention group. Attrition bias will be minimised by having robust trial procedures to prevent data loss and also analysing the data by intention to treat (ITT). |
| 17b | Circumstances under which unblinding is permissible, and procedure for revealing a participant’s allocated intervention during the trial | The DMEC will undertake safety data reviews every 12 months after recruitment begins, and all SAEs will be reported to them. The DMEC will be responsible for identifying any need for unblinding. The DMEC will also periodically reviewing unblinded overall safety data to determine patterns and trends of events, or to identify safety issues, which would not be apparent on an individual case basis. |
| 18a | Data collection methods | Completed CRFs will be checked and signed at the assessment sites by a member of the research team before being taken to the local research site. Data from the original CRF pages and SPPB result forms will be entered on to a password-protected website designed and maintained by the Peninsula Clinical Trials Unit. All CRF pages and data collection forms will be tracked using the website. |
| 18b | Plans to promote participant retention | Loss to follow up is modelled on an annual attrition rate of 12.5%. This is based on attrition rates in the Better Ageing study and LIFE (6.5% per year) which were both community-based physical activity intervention studies. The internal pilot study will demonstrate that recruitment and retention rates are satisfactory and established at each site before we progress to the full-scale trial. To maximise retention, we will offer a voucher-based incentive for trial completion (one of the most effective strategies identified by a recent Cochrane Review^66^) and we will follow recommendations for good practice for retention in trials provided by the NIHR School for Primary Care Research^80^. These include emphasising the meaningfulness of the research, regular contact, use of incentives and involving service users in development of study materials, actively building social networks, the Ambassador’s programme and supporting the engagement of participants in community activities. |
| 19 | Plans for data entry, coding, security, and storage | Data from the original CRF pages and SPPB result forms will be entered on to a password-protected website designed and maintained by the Peninsula Clinical Trials Unit. All CRF pages and data collection forms will be tracked using the website. Double-entered data will be compared for discrepancies using a report available on the website. Discrepant data will be verified using the original paper data sheets and incorrect values will be updated. Audit trails will be used to record all change to study data. Accelerometer data and data from the computerised UK Biobank Healthy Minds Questionnaire will be imported directly into the study database at each site. Anonymised brain imaging data and data from detailed cognitive assessments for the fMRI imaging sub-study will be stored on password protected secure servers at the Universities of Bristol and Oxford.  **Data Confidentiality**  Participant names and addresses will be collected for the purpose of managing questionnaires, intervention delivery and process evaluation interviews. Investigators will ensure that the participants’ anonymity is maintained on all other documents. Within each trial site, anonymised and identifiable study data will be stored separately, to prevent the identification of participants from research records, in locked filing cabinets within a locked office. Electronic records will be stored at each site in a SQL server database, housed on a restricted access, secure server. Data in the database will be backed up daily by IT services at the Universities of Bath, Birmingham and Exeter. Back-ups will be accessible for up to 6 months. The website will be encrypted using SSL. Anonymised brain imaging data and data from detailed cognitive assessments for the fMRI imaging sub-study will be stored on password protected secure servers at the Universities of Bristol and Oxford. Data will be collected and stored in accordance with the Data Protection Act 1998. Direct access to the trial data will be restricted to members of the research team, with access granted to the Sponsor on request. Access to the database will be overseen by the CI and trial coordinator. Copies of original study data retained at trial sites will be securely stored for the duration of the study prior to archiving. Audio recordings and participant names and addresses will be stored on a restricted access, secure servers at the Universities of Bath, Birmingham and Exeter.  All data entered into the website provided by the Peninsula Clinical Trials Unit will be stored in a SQL Server database, housed on a restricted access, secure server managed by Plymouth University.  Data in the database will be backed up daily by IT services at Plymouth University. Back-ups will be available for the entire duration of the study. All data transferred to and from the website to the SQL Server database will be encrypted using SSL. Data will be collected and stored in accordance with the Data Protection Act 1998. Direct access to the trial data will be restricted to members of the research team, with access granted to the Sponsor on request. Access to the website will be overseen by the CI and trial coordinator. |
| 21a | Composition of data monitoring committee (DMC) | A fully independent DMEC has been appointed which will report to the TSC. This will comprise of an independent chair Professor Dawn Skelton (Glasgow Caledonian University), Professor Paul Ewings, the Director of NIHR Research Design Service South West and Professor Kamlesh Khunti, Professor in Primary Care (University of Leicester). The CI, PIs and Chief trial statistician may be invited to attend to provide specific input by the DMEC Chair with the CI and statistician usually expected to attend the ‘open session’ section of the meetings. The DMEC will be responsible for the interests of the participants and its main role will be to make recommendations to the TSC as to whether the trial needs to be stopped for any ethical or safety reason (based on review of accumulating safety data). The DMEC will undertake safety data reviews every 12 months after recruitment begins, unless otherwise deemed necessary. This will include data on any adverse advents reported during the trial. Analysed data will be blinded, unless the DMEC identifies a specific need for unblinding. |
| 21b | Description of any interim analyses and stopping guidelines | The Trial Steering Committee, with advice from the Data Monitoring and Ethics Committee, will assess the feasibility of the trial during the internal pilot phase, taking into account findings on the acceptability of trial procedures, intervention adherence and recruitment and retention rates. Based on our recruitment rates in previous UK-based physical activity interventions with similar target populations (Project ACE, Better Ageing) and with equivalent of 1FTE research assistants at each of three sites, in the pilot study we anticipate a recruitment rate of 15 participants/month/site, (180 participants will be recruited in total over 4 months). If the recruitment rate is less than predicted in a given month, we will take actions to increase it (increasing the number of people approached and/or increasing the geographical area, adapting recruitment procedures). After 6 months, recruitment data will be reviewed by the TSC and any required changes in the recruitment strategy and/or introduction of new recruitment avenues will be discussed and agreed. Retention rates (proportion of people providing follow up data) will also be checked at 6 months. Receipt of strong negative feedback from the majority of either participants or intervention providers about the intervention or trial methods will be considered as a stopping criterion. The participants recruited in the pilot study will be included in the trial analysis. |
|  |  | **Recording and reporting of SAEs**  The definitions of the EU Directive 2001/20/EC Article 2 based on the principles of ICH Good Clinical Practice will apply. The University of Bath standard operating procedure for reporting research related Adverse Events (AEs) will be adopted*.*  Adverse Event (AE) is any untoward medical occurrence, unintended disease or injury or any untoward clinical signs in subjects, users or other persons whether or not related to any research procedures or to the intervention.  Non-serious adverse events which are not related to study procedures or to the intervention will **not** be reported in this study.  The expression ‘reasonable causal relationship’ means to convey, in general, that there is evidence or argument to suggest a causal relationship. PIs or Research Assistants will assess the causal relationship between reported events and trial participation according to the standardised guidance given below:   | **Table 2 Causal relationship between reported events and trial participation** | | | --- | --- | | **Relationship** | **Description** | | Unrelated | There is no evidence of any causal relationship | | Unlikely | There is little evidence to suggest there is a causal relationship (e.g. The event did not occur within a reasonable time after the study period). There is another reasonable explanation for the event (e.g. The participant’s clinical condition, other concomitant treatment). | | Possible | There is some evidence to suggest a causal relationship (e.g. Because the event occurs within a reasonable time after the study period) However, the influence of other factors may have contributed to the event (e.g. The participant’s clinical condition, other concomitant treatments). | | Probably | There is evidence to suggest a causal relationship and the influence of other factors is unlikely. | | Definitely | There is clear evidence to suggest a causal relationship and other possible contributing factors can be ruled out. |   **Seriousness**  Any adverse event or adverse reaction will be regarded as serious if it:  i. results in death;  ii. is life threatening;  iii. requires hospitalisation or prolongation of existing hospitalisation;  iv. results in persistent or significant disability or incapacity  Therefore, an adverse event meeting any one of these criteria will be a **Serious Adverse Event (SAE).** In this study, all serious events will be reported regardless of relatedness. Any non-serious adverse events (regardless of relatedness) will not be reported. All reportable events will be followed until resolution where possible or until the end of the data collection period.  **Reportable events**   | **Table 3 *-* Reportable adverse events** | | | | | --- | --- | --- | --- | | Event type | Reported by | Reported to | Timeframe | | Serious Adverse Event (SAE) | Local PI, TM or RA | CI, DMEC, Sponsor | Within 24 hours* |  |  | - *To the of the DMEC by PI (or authorised delegate)* | | | --- | --- | --- | |  | |  |  Notification of deaths All deaths, including deaths deemed unrelated to the trial, if they occur earlier than expected will be reported to the DMEC immediately.  Following up SAEs (where data missing or event not resolved)  Where there is missing data/queries or the event is not yet confirmed as resolved, the RA will manage the event/chase the data until the form is complete. RA will update the database with all new information received. When the SAE form is complete the RA will file the SAE form in the site file. Development safety update reports The CI will provide DSURs once a year throughout the trial, or on request, to the Ethics Committee and Sponsor. |
| 23 | Frequency and procedures for auditing trial conduct | Participating sites will be required to permit a representative of the TSC or representative of the sponsor, to undertake study-related monitoring to ensure compliance with the approved study protocol and applicable SOPs, providing direct access to source data and documents as requested.  All study procedures will be conducted in compliance with the protocol and according to the principles of the International Conference on Harmonisation Good Clinical Practice (ICH GCP). Procedures specifically conducted by the CTU team (e.g. randomisation) will be conducted in compliance with CTU standard operating procedures (SOPs). |
| 25 | Plans for communicating important protocol modifications | Any amendments to the protocol will be submitted for REC approval as appropriate. Substantial amendments that require review by REC will not be implemented until the REC grants a favourable opinion for the study. |
| 27 | Confidentiality | Participant names and addresses will be collected for the purpose of managing questionnaires, intervention delivery and process evaluation interviews. Investigators will ensure that the participants’ anonymity is maintained on all other documents. Within each trial site, anonymised and identifiable study data will be stored separately, to prevent the identification of participants from research records, in locked filing cabinets within a locked office. Electronic records will be stored at each site in a SQL server database, housed on a restricted access, secure server. Data in the database will be backed up daily by IT services at the Universities of Bath, Birmingham and Exeter. Back-ups will be accessible for up to 6 months. The website will be encrypted using SSL. Anonymised brain imaging data and data from detailed cognitive assessments for the fMRI imaging sub-study will be stored on password protected secure servers at the Universities of Bristol and Oxford. Data will be collected and stored in accordance with the Data Protection Act 1998. Direct access to the trial data will be restricted to members of the research team, with access granted to the Sponsor on request. Access to the database will be overseen by the CI and trial coordinator. Copies of original study data retained at trial sites will be securely stored for the duration of the study prior to archiving. Audio recordings and participant names and addresses will be stored on a restricted access, secure servers at the Universities of Bath, Birmingham and Exeter.  All data entered into the website provided by the Peninsula Clinical Trials Unit will be stored in a SQL Server database, housed on a restricted access, secure server managed by Plymouth University.  Data in the database will be backed up daily by IT services at Plymouth University. Back-ups will be available for the entire duration of the study. All data transferred to and from the website to the SQL Server database will be encrypted using SSL. Data will be collected and stored in accordance with the Data Protection Act 1998. Direct access to the trial data will be restricted to members of the research team, with access granted to the Sponsor on request. Access to the website will be overseen by the CI and trial coordinator. |
| 29 | Access to data | Direct access will be granted to authorised representatives from the Sponsor, host institutions and the regulatory authorities to permit trial-related monitoring, audits and inspections.  Prior to the first report/publication being made (the publication(s) reporting the results of the research as a whole), the collaborators cannot report on the results (those collected at their site and from the project as a whole) without first gaining consent from the CI. Thereafter the collaborators can independently publish the results subject to provisions of confidentiality.  The NIHR’s Policy on Open Access will be adhered to and data supporting published findings will be made accessible. Subject to data protection provision (data to be anonymised), the Secretary of State for Health has the right to have access and use data collected and used for the purpose of the project. |
| 30 | Ancillary and post-trial care and compensation to those who suffer harm | The University of Bath has arranged Public Liability insurance to cover the legal liability of the University as Research Sponsor in the eventuality of harm to a research participant arising from management of the research by the University.  The University of Bath holds Professional Indemnity insurance to cover the legal liability of the University as Research Sponsor and/or as the employer of staff engaged in the research, for harm to participants arising from the design of the research, where the research protocol was designed by the University.  The University of Bath’s insurance policies do not provide an indemnity to collaborators. As Research Sponsor we will ensure as far as reasonably practicable at the outset of the study that collaborators hold appropriate legal liability insurance.  The University of Bath has not made arrangements for payment of compensation in the event of harm to the research participants where no legal liability arises. |
| 31a | Dissemination policy | Research findings will be disseminated using several channels to ensure maximum exposure:  1. Our OPAL and AVONet websites will be updated to include a section for publishing REACT news and progress. All research presentations and reports will be uploaded and made available for public comments;  2. A one day launch event will be co-hosted with the partner organisations.  3. Showcase events will be delivered at all three sites after completion of REACT to present the findings and celebrate successful lifestyle change stories;  4. At least 5 papers will be submitted for publication in peer reviewed journals, including open access journals (e.g., International Journal of Behaviour Nutrition & Physical Activity), subject-specific journals (e.g., Journal of the American Geriatrics Society) and medical journals (e.g., Annals of Behavioural Medicine, NIHR PHR Journal);  5. Presentations will be delivered at University-sponsored Public Lecture series, national (UK Society of Behavioural Medicine) and International (World Congress of Aging and Physical Activity) conferences, and events organised by local partner organisations;  6. Newsletters will be distributed to participants at the end of each project year and through academic and non-academic partners and the European Network for Action on Ageing and Physical Activity;  7. Social media (including Universities’ Twitter accounts and Facebook pages) and local media (newspapers, magazines) will be used to publish news briefings prepared by the Universities’ press offices;  8. We will capitalise on the extensive distribution channels of AGE UK to disseminate information in a more visual and user friendly way, and via the Action Age Alliance which involves over 570 partner organisations including eight Government Departments and representatives from public, private and voluntary sectors. Our MRC funded work has already been disseminated via Action Age Alliance (<http://ageactionalliance.org/wordpress/wp-ontent/uploads/2014> /03/AVONetreport-2014-March.pdf) and we will continue this successful collaboration. |
| 31b | Authorship eligibility guidelines and any intended use of professional writers | The International Committee of Medical Journal Editors’ authorship criteria (detailed below) will be used as the basis for granting authorship of the REACT final trial report.   - Substantial contributions to the conception or design of the work; or the acquisition, analysis, or interpretation of data for the work; AND - Drafting the work or revising it critically for important intellectual content; AND - Final approval of the version to be published; AND - Agreement to be accountable for all aspects of the work in ensuring that questions related to the accuracy or integrity of any part of the work are appropriately investigated and resolved.   A detailed publication plan with proposed authorship will be developed and agreed by the TMG during the first year of the Trial.  Professional writers will not be used in the development of the REACT trial reports |
| 31c | Plans for granting public access to the full protocol, participant-level dataset, and statistical code | The NIHR’s Policy on Open Access will be adhered to and data supporting published findings will be made accessible.  Subject to data protection provision (data to be anonymised), the Secretary of State for Health has the right to have access and use data collected and used for the purpose of the project. |
