# Supplementary material for: A community-based physical activity intervention to prevent mobility-related disability for retired older people (REtirement in ACTion (REACT)): study protocol for a randomised controlled trial
Source: Trials. 2018 Apr 17;19:228. doi: 10.1186/s13063-018-2603-x (PMC5905123; doi:10.1186/s13063-018-2603-x)
Supplement: Supplementary file 3 — REACT process evaluation protocol and Logic model. (DOCX 128 kb) [file 13063_2018_2603_MOESM3_ESM.docx]

**REACT: REtirement in ACTion**

**PROCESS EVALUATION PROTOCOL**

Version: 4 25^th^ May 2017

Trial registry number and date ISRCTN 45627165 13/06/2016

Project Reference Number: 13/164/51

Study Sponsor: University of Bath

Chief Investigator: Dr Afroditi Stathi (University of Bath)

This document is a process evaluation plan for the REACT randomised controlled trial. In developing this document, account has been taken of the recommendations outlined in the MRC guidance on process evaluation.

Section 1 outlines key points from the MRC guidance that have been considered. Section 2 describes a logic model for REACT which provides a basis for the process evaluation. Section 3 provides the hypotheses tested in the process evaluation. Sections 4 and 5 summarise plans for the process evaluation, including both qualitative and quantitative data collection. Section 6 provides the topic guides for the qualitative evaluation and section 7 provides a list of references.

**1. Background: The MRC process evaluation guidance**

MRC process evaluation guidance advises that the starting point to design a process evaluation is to clearly specify the causal assumptions underlying the intervention and its implementation. That is, a clearly articulated logic model is required at the outset. This will enable the process evaluation to investigate the plausibility of the logic model by examining the relationships specified.

The purposes of process evaluation in the REACT trial are to:

- evaluate the feasibility of (barriers to and facilitators of) implementation to inform future implementation and possible refinements of the intervention
- evaluate the quality and quantity of intervention delivery to inform conclusions about intervention effectiveness
- investigate the proposed mechanisms of change, outlined in the logic model and to seek alternative explanations if this model is not supported
- understand the role of context to inform whether and how the findings can be generalised

**1.1 Content of process evaluations**

**Fidelity, dose**

An intervention might not 'work' because it was not well designed or because it was not properly implemented. It might work, even if it was not implemented as intended. Therefore, a process evaluation should capture:

- whether the intervention was delivered as intended (fidelity)
- how much of the intervention was delivered (dose)

Adaptations can be made to the implementation of an intervention which enables it to fit different contexts and a process evaluation can help identify which of these adaptations might undermine or enhance effectiveness.

**Delivery methods**

Understanding the means of delivering an intervention through process evaluation can inform how the intervention can be replicated in the 'real world'. These means include the provision of training, communication, management, programme delivery, attitudes of service providers and managers and the relationship between these factors.

**Reach**

Process evaluation can also investigate whether and how participants come into contact with the intervention and how generalisable the intervention is (or is likely to be) in different contexts.

**Mechanisms**

To understand the causes of effects from an intervention requires identification of the mechanisms by which change is achieved. Process evaluation can test whether the theory about an intervention’s causal pathways (as articulated by a logic model) 'works'.

**Context**

Factors external to the intervention can potentially act as a barrier or facilitator to implementation or to its effects. Implementation or mechanisms might also need to be adapted to these contexts to enable the intervention to work. Equally, implementation might not vary but the effect of the intervention will vary depending on context. Process evaluation can be used to understand contexts and their relationship with implementation, mechanisms and effects (e.g. whether ethnicity moderates intervention effectiveness).

**Process evaluation research questions**

It is not realistic for a process evaluation to address all aspects of the implementation of an intervention. It is preferable to provide satisfactory answers to the most important questions than to inadequately address too many questions. To identify the key questions, it is necessary to identify the causal assumptions of the intervention model and which of these have the most limited evidence base. Further research questions might arise during the implementation process - therefore a process evaluation should be flexible in order to respond to emerging questions.

**1.2 Implications of MRC guidance for the REACT process evaluation**

In developing the plan to carry out a process evaluation of REACT, clarity is needed about:

- The purposes of the process evaluation (research questions)
- The logic model and which elements of it that the process evaluation will address
- Which elements of implementation the process evaluation will address (e.g. fidelity, dose, delivery methods, reach)
- The extent to which context and mechanisms will be investigated
- The methods required to address the research questions

The remainder of this plan covers these issues.

**2. The REACT logic model**

**2.1: Theoretical basis for the REACT intervention**

The REACT intervention comprises and exercise class and social education sessions. The REACT exercise classes are structured and include instruction and practice of muscle-strengthening /function-sustaining exercises that are adapted for each individual’s functional abilities, aiming to progress these gradually over time. Despite the relative formal structure, exercise facilitators are expected to encourage fun and social interaction during the sessions. They are also trained to gather feedback and promote /discuss participants ideas about “transition” to using similar exercises in their day to day lives when the REACT sessions reduce in frequency (which happens at 12 weeks and at 12 months). The REACT social /education programme is more informal and aimed at maximising social interaction and enjoyment. However, it will contain some structured elements designed to teach participants skills that will help them to plan, implement and maintain a healthy level of physical activity.

The REACT programme draws on the following, overlapping (and mutually compatible) theoretical perspectives. Social Cognitive Theory and Self Determination Theory provide the main principles and processes for supporting behaviour change. The Skills for Maintenance (SkiM) model (below) has been used to identify additional processes and techniques to promote maintenance of physical activity /exercise.

*Social Cognitive Theory*[1, 2]

People can learn by observing others and the consequences of their actions, as well as by getting feedback on their own actions. Learners can acquire new behaviours and knowledge by observing and copying a model (another person), especially if they identify positively with the model. This overlaps to some extent with the concept of “relatedness” in Self-Determination Theory (below).

People set goals for themselves based on outcome expectancies (expectation of benefit) and self-efficacy (perceived ability to achieve the behaviour) and direct their behaviour accordingly. They are then motivated to continue a new activity to the extent that they get positive feedback about a) benefits and dis-benefits of doing the activity (outcomes) and b) their experienced ability to master /achieve the new behaviour (self-efficacy).

The process of behaviour change /maintenance is cyclic, with positive outcomes and the building of self-efficacy, as well as environmental factors acting to reinforce continuation. This process of self-regulation or “learning from experience” requires time for learning of new behaviours to be embedded.

*Self-Determination Theory (SDT)[3-5]*

SDT proposes that three psychological needs motivate people to initiate and sustain behaviour. These needs are held to be universal and innate and include the need for [competence](https://en.wikipedia.org/wiki/Competence_(human_resources)) (feeling capable and confident), [autonomy](https://en.wikipedia.org/wiki/Autonomy) (feeling in control of decisions /goals, having motivation that is intrinsic (self-generated)), and [relatedness](https://en.wikipedia.org/wiki/Social_relation) (social engagement, social acceptance /approval of the behaviour, giving support to others). Fulfilling these needs through engagement in a social physical activity programme may lead to an improvement in the social and control /competence domains of the self-concept.[6]

*The Skills for Maintenance (SkiM) model*[7]

This new model focuses on the skills that people need to maintain lifestyle changes and to overcome challenges to Physical, Psychological and Social needs that are generated by changes in lifestyle. The main premise is that changing your lifestyle can induce physical, psychological and social tensions in your life. The sources of tension for physical activity may include conflict with other priorities in life, conflict with the needs of others, discomfort /lack of enjoyment associated with the new activity, conflict with established habits, or conflict with established beliefs and attitudes (including self-concept or “identity” beliefs). This tension can be managed in the short term through willpower, self-regulation and regular re-motivation, as well as by making plans to manage any slips and lapses that occur. However, to achieve long-term change requires individuals to either pre-empt and prevent the tension (make changes you can live with /will enjoy), or to resolve it by finding other ways to address the sources of the tension (e.g. negotiating to resolve conflicts with the needs of others; actively challenging unhelpful thoughts and beliefs). Learning from experience and eventual change in self-concept (particularly the physical, social, emotional and control /competence self-concept domains of self-concept) are hypothesised to be important determinants of long-term success. The power of relatedness (from self-determination theory) is acknowledged, but the need to ensure that change is embedded in social support within day-to-day life and not dependent on temporary relationships formed in intervention group settings is an important consideration. It is proposed that the processes of planning, self-regulation, making psychological changes and social interactions to address sources of tension and achieve long-term lifestyle change are teachable skills and techniques have been developed to facilitate this. The model provides a way of ensuring a clear focus on the challenge of long-term maintenance and has been used to the extent that it adds to or extends the above theories. Specifically, this includes the use of techniques to promote habit formation, to plan for sustainability (of social support relationships as well as behaviours), to address hedonic needs, to address priority conflicts and to recognise and reinforce benefits in terms of self-concept change (particularly in the physical, emotional, social and competence domains).

**2.2: Logic model**

The logic model for REACT is shown in Figure 1. It identifies:

- The REACT intervention components and how they are delivered to participants
- The hypothesised mechanisms of action of the REACT intervention (causal assumptions about the process by which the intervention effects change in health behaviours and outcomes)
- The hypothesised contextual variables which might affect mechanisms of change in motivations and behaviour
- The hypothesised interaction between participation in the intervention, delivery quality, motivation, behaviour and outcomes.

The logic model also shows the types of data that will be collected for the process evaluation during the trial.

**INTERVENTION COMPONENTS /PROVIDER TRAINING**

REACT is designed to help participants to use physical activity to maximise a) functional abilities and b) quality of life:

1: Exercise programme to build lower limb muscle strength and CV fitness

2: Social interaction to maximise enjoyment and motivation to continue participation

3. Group-based activities designed to build and maintain intrinsic motivation, plan more physical activity in peoples’ day-to-day lives, identify and solve problems and build competence

4. Monitoring progress in activity levels and perceived emotional, physical and social benefits, to sustain motivation for PA (and attendance).

5. Person-centred delivery style to build autonomy /intrinsic motivation.

6. A strong focus on maintenance through building sustainable support networks, teaching techniques for managing slips /lapses, supporting habit change and identifying and resolving sources of tension around increasing physical activity.

NB – These are the key components for monitoring delivery quality.

Qualitative: Interviews with trainees to assess receipt of key principles and extract ideas to improve training.

**CONTEXT**

Participant engagement with REACT (attendance), PA and SPPB outcomes at 6,12,24 mth may be moderated by:

- Participant characteristics (Age, Gender, ethnicity, Baseline SPPB /PA, Mental health, SES, Education)
- Site, intervention provider organisation, coach, relationship with REACT coach
- Co-interventions and co-morbidities (inc. BMI)

Quantitative: Data on contextual factors collected through baseline CRF /questionnaires from intervention and control group.

Qualitative: Data on contextual factors collected through interviews with intervention group

**LONG TERM OUTCOMES**

PA and SPPB change maintained, QoL increases

Heath economic benefits

Maintenance of PA change at 12,24 mth is moderated by perceived “tension” at 6 and 12 mth. And also by autonomy, competence, relatedness, enjoyment of PA, the perceived benefits of changes in PA (social, physical, emotional) and positive changes in social and physical self-concept.

Change in SPPB /disability outcomes due to intervention mediated by exercise and potentially physical activity.

Quantitative: Evaluation of outcomes via questionnaire and accelerometer. Process analyses to test moderation & mediation hypotheses.

**SHORT TERM OUTCOMES**

Intervention increases PA and SPPB at 6,12, 24 mth. This is mediated by changes in autonomy, competence and relatedness (within and external to group), and moderated (in I group) by enjoyment and perceived benefits of PA (social, physical, emotional).

Attendance of the programme is moderated by enjoyment of the programme and perceived benefits (social, physical and emotional benefits).

Quantitative: Questionnaires measuring autonomy, competence and relatedness (relating to PA), enjoyment and perceived benefits of the programme at 6,12 mth.

Qualitative: Data on participant experiences, motivations, sources of tension and changes collected through interviews with intervention group at 6, 12 mth.

**INTERVENTION DELIVERY**

Facilitators guide participants through the REACT programme.

Quantitative: Session recordings coded to assess delivery quality and “receipt”. Group engagement, receipt and “enactment” assessed by 6,12 mth questionnaire

Qualitative: Interviews with intervention group to assess participant experience of REACT.

**FEEDBACK LOOPS**

Participant attendance, use of BCTs, increases in PA are reinforced by perceptions of benefit (emotional, physical and social), as well as enjoyment of the programme (and PA), positive changes in social and physical self-concept and perceived autonomy, competence and relatedness (for PA). These interactions can build into positive cycles of perceived benefit and behaviour change, but may be mediated by delivery quality and perceived tension.

Qualitative: To explore positive or negative feedback loops and other interactions via interviews with intervention group at 6,12 mth.

**Fig. 1: REACT logic model (and associated data collection)**

**3. Hypotheses for process evaluation of the REACT intervention**

The hypotheses below are derived from the stated and implied assumptions in the above logic model and will be tested using process and outcomes data collected at baseline, 6 months (short term), 12 months (medium term) and 24 months (long term). The time from baseline to 12 months represents the intervention period (0-6 months = initial change and 6-12 months = supported maintenance) and the 12-24 month timeframe represents the post-intervention (unsupported maintenance) period.

*Effects of the intervention on mediators of (and change in) lower limb physical function*

REACT Intervention (Yes /No)

PA

Exercises

Better SPPB scores

1. Being in the intervention group will lead to changes in physical activity (MVPA, steps, sedentary time) and engagement in muscle-strengthening exercise from 0 to 6 months. This will be tested by comparison of change scores between intervention and control groups. Baseline values and other possible covariates (see main trial analysis plan) will be included in the model.
2. Increased exposure to the intervention (contact time) will be associated with increased change in physical activity (MVPA, steps, sedentary time) and engagement in muscle-strengthening exercise from 0-6 months. This will be tested by within group regression modelling, with due attention to possible covariates /moderating factors.
3. Increased exposure to the intervention (contact time) will be associated with increased change in physical activity (MVPA, steps, sedentary time), engagement in muscle-strengthening exercise and SPPB score from 0-12 months. This will be tested by within group regression modelling, with due attention to possible covariates /moderating factors.
4. Increased exposure to the intervention should lead to increased maintenance of physical activity and engagement in exercise. So, within the intervention group, intervention dose (contact time) will be negatively associated with decreases in PA, engagement in muscle-strengthening exercise and SPPB score from 6-12 months (during the supported maintenance period) and from 12-24 months (the unsupported maintenance period). This will be tested by within group regression modelling, with due attention to possible covariates /moderating factors.

*Effects of the intervention on mediators of PA and exercise*

REACT Intervention (Yes /No)

Determinants of PA /exercise

PA

Exercises

1. Exposure to the intervention should lead to changes in key psychosocial determinants (specified in the theories underpinning the intervention) of physical activity and exercise from baseline to 6 and 12 months.
   1. Compared with controls, the intervention group will experience increases in autonomy, competence (self-efficacy), relatedness, perceived intrinsic benefits of PA and exercise (social, physical and emotional) and enjoyment of PA and exercise from 0-6 months. This will be tested by comparison of change scores between intervention and control groups. Baseline values and other possible covariates (see main trial analysis plan) will be included in the model.
   2. Compared with controls, the intervention group will experience increases in physical activity-related self-concept, autonomy, competence (self-efficacy), relatedness, perceived intrinsic benefits of PA and exercise (social, physical and emotional) and enjoyment of PA and exercise from 0-12 months.
   3. Increased exposure to the intervention (total contact time from baseline to the relevant time point) will be associated with increased change in the above determinants (and in the expected direction). This will be tested by within group regression modelling, with due attention to possible covariates.

*Mediation and moderation of intervention effects on lower limb physical function*

1. The intervention effect on SPPB (I vs C) from 0-6, 0-12 and 0-24 months may be mediated by changes in muscle-strengthening exercise, changes in balance and co-ordination exercise and by changes in MVPA, changes in lower intensity PA, or walking activity (steps per week). The amount of variance in SPPB explained by the different types of activity /exercise will be of interest. This will be tested by exploratory mediation analyses.
2. The intervention effect on change in SPPB score from 0-6, 0-12, 0-24 months may be moderated by a number of potential moderating variables, including Age, Gender, Ethnicity, Baseline physical activity and SPPB, Co-interventions, Co-morbidities, BMI, Mental health, Socio-economic status, Education level. This will be tested by exploratory moderation analyses.
3. The intervention effect on *maintenance* of SPPB score from 6-12 and 12-24 months may be moderated by a number of potential variables, including Age, Gender, Ethnicity, Baseline physical activity and SPPB, Co-interventions, Co-morbidities, BMI, Mental health, Socio-economic status, Education level. This will be tested by exploratory moderation analyses.

*Mediation and moderation of intervention effects on physical activity and exercise*

1. Intervention effects on PA and muscle-strengthening exercise at 6 and 12 months will be mediated by 0-6 mth changes in autonomy, competence and relatedness in relation to PA and exercise, enjoyment of PA and exercise and perceived intrinsic benefits of PA and exercise (social, physical and emotional). This will be tested by pre-specified mediation analyses.
2. The intervention effect on PA and exercise at 6 and 12 months may be moderated by a number of potential moderating variables, including Age, Gender, Ethnicity, Baseline physical activity and SPPB, Co-interventions, Co-morbidities, BMI, Mental health, Socio-economic status, Education level. This will be tested by exploratory moderation analyses.
3. For those who succeed in increasing PA and exercise from 0 to 6 months (defined as an increase of at least 45 mins per week of MVPA), the amount of change in PA maintained from 6 to 12 months (supported maintenance) will be associated with perceived autonomy, competence and relatedness for PA and exercise, enjoyment and perceived intrinsic benefits of PA and exercise (social, physical and emotional) at 6 months (NB: the predictor here is absolute values at 6 mths, not change scores). This will be tested by within group regression modelling, with due attention to possible covariates.
4. For those who succeed in increasing PA and exercise from 0 to 12 months (defined as an increase of at least 45 mins per week of MVPA), the amount of change in PA maintained from 12 to 24 months (unsupported maintenance) will be associated with perceived autonomy, competence and relatedness for PA and exercise, enjoyment of and perceived intrinsic benefits of physical activity and exercise (social, physical and emotional) at 12 months (NB: the predictor here is absolute values at 12 mths, not change scores), as well as by change in physical activity related self-concept from 0-12 months. This will be tested by within group regression modelling, with due attention to possible covariates.
5. For those who succeed in increasing PA and exercise at 6 months (defined as an increase of at least 45 mins per week of MVPA), the amount of change in PA from 6 to 12 months (supported maintenance) will be associated with perceived “tension” (see SkiM theory description for definition) of making changes in PA and exercise at 6 months. This will be tested by within group regression modelling, with due attention to possible covariates. The analysis may need to be controlled for amount of PA and exercise increase (0-6 mths) as more extreme changes in PA or exercise should induce higher tension.
6. For those who succeed in increasing PA and exercise at 12 months (defined as an increase of at least 45 mins per week of MVPA), the amount of change in PA from 12 to 24 months (unsupported maintenance) will be associated with perceived tension of making changes in PA and exercise and changes in physical activity related self-concept at 12 months. This will be tested by within group regression modelling, with due attention to possible covariates. The analysis may need to be controlled for amount of PA and exercise increase (0-12 mths) as more extreme changes in PA or exercise should induce higher tension.
7. For those who succeed in increasing PA and exercise at 6 and 12 months (as defined above), low tension participants will have increased enjoyment of PA and exercise and a more positive physical activity related self-concept than higher tension participants at 6, 12 and 24 months (cross-sectionally and potentially prospectively also). This will be tested by within group regression modelling, with due attention to possible covariates.

*Mediators and moderators of programme attendance*

1. Within the intervention group, programme attendance (contact time, number of sessions attended) from 0-6 months will be associated with enjoyment of the programme, positive perceptions of the facilitators and perceived benefits of PA and exercise (social, emotional, physical) at 6 months (absolute value, rather than change score). This will be tested by within group regression modelling, with due attention to possible covariates.
2. Within the intervention group, programme attendance (contact time, number of sessions attended) from 0-12 months will be associated with enjoyment of the programme and perceived benefits of PA and exercise (social, emotional, physical) at 6 months (absolute values, rather than change scores). This will be tested by within group regression modelling, with due attention to possible covariates.

*Mediators and moderators of people joining the ambassadors programme*

1. Within the intervention group, engagement with the ambassador programme (Yes /No) at 12 months will be associated with enjoyment of the programme, positive perceptions of the facilitators, relatedness in relation to PA and exercise (combined) and perceived benefits of PA and exercise (social, emotional, physical) at 6 months, and change in physical activity related self-concept (particularly social self-concept) from 0-12 months. This will be tested by within group logistic regression modelling, with due attention to possible covariates.

**Additional considerations:** Delivery style (and other engagement processes) and intervention fidelity may also moderate the effectiveness of the intervention. However, these concepts cannot be measured at the individual level and will only be assessed (through researcher observation of sessions) for a sub-sample of intervention sessions. These hypotheses may also be explored qualitatively.

**4. Quantitative process evaluation**

**4.1 Participants /sampling**

We will apply analyses to the whole sample where data is available, unless otherwise stated (e.g. some hypotheses apply only within the intervention group).

**4.2 Measures**

The following will be measured using facilitators’ records of each session and brief questionnaires (given to participants at each assessment point) to allow testing of the above hypotheses:-

- Session attendance
- Total contact time for each participant
- Muscle strength /balance (SPPB scores)
- Physical activity (accelerometry) - time doing MVPA in bouts of either 1 or 10 minutes, steps, sedentary time)
- Engagement in muscle-strength exercise
- Physical activity related self-concept
- Perceived tension of maintaining current PA
- Perceived tension of maintaining current levels of exercise
- Autonomy in relation to PA
- Competence for PA
- Relatedness for PA
- Enjoyment of PA
- Perceived intrinsic benefits of PA (social, physical and emotional)
- Autonomy for strength-building exercise
- Competence for strength-building exercise
- Relatedness for strength-building exercise
- Enjoyment of strength-building exercise
- Perceived intrinsic benefits of strength-building exercise (social, physical and emotional)
- Enjoyment of the REACT programme (I group only)
- Credibility /identification with the session facilitators (I group only)
- *Demographic variables:* Age, Gender, Ethnicity, Baseline physical activity and SPPB, Co-interventions (0.6.12.24), Co-morbidities, BMI, (0,6,12,24), Mental health, Multiple Deprivation Index (from postcode), Education level.

|  |  |  |  |  |  |
| --- | --- | --- | --- | --- | --- |
| **Visit type** | **Scr** | **Scr** | **FU** | **FU** | **FU** |
| **Visit code** |  | **SV1** | **F06** | **F12** | **F24** |
| Visit number |  | 1 | 2 | 3 | 4 |
| Telephone call | 1 |  |  |  |  |
| Activity/assessment Month | -0.5 | 0 | 6 | 12 | 24 |
| **Form Name** |  |  |  |  |  |
| Verbal consent | X |  |  |  |  |
| Telephone screening (some elements of inclusion and exclusion criteria) | X |  |  |  |  |
| Written informed consent |  | X |  |  |  |
| Contact information update | X | X | X | X | X |
| Demographic, social, economic | X |  |  |  |  |
| SPPB battery |  | X | X | X | X |
| Accelerometry |  | X | X | X | X |
| Engagement in muscle-strength exercise (questionnaire) |  | X | X | X | X |
| Height and weight |  | X |  |  | X |
| MoCA – Montreal Cognitive Assessment |  | X | X | X | X |
| PASE questionnaire |  | X | X | X | X |
| Dynometer (hand grip strength) |  | X | X | X | X |
| Ageing Well profile (social well-being scale only) |  | X |  | X | X |
| Health-related quality of life (EQ-5D, SF-36) |  | X | X | X | X |
| Sleep Condition Indicator |  | X |  | X | X |
| Pain (Western Ontario and McMaster Universities Osteoarthritis Index (WOMAC) |  | X |  | X | X |
| Mobility assessment tool-short form (MAT-sf) |  | X | X | X | X |
| Cognitive function (UK Biobank Healthy Minds Questionnaire) |  | X | X | X | X |
| Medical history |  | X |  |  |  |
| Falls Inventory |  | X | X | X | X |
| Health and Social Service Usage |  | X | X | X | X |
| *(fMRI imaging substudy)* MRI scan, detailed cognitive assessment and gait analysis |  | X | X | X |  |
| Session attendance (Intervention group only)  Total contact time for each participant  Physical activity related self-concept  Perceived tension of maintaining current PA  Perceived tension of maintaining current exercise  Autonomy in relation to PA  Competence for PA  Relatedness for PA  Enjoyment of PA  Perceived intrinsic benefits of PA (social, physical and emotional)  Autonomy for strength-building exercise  Competence for strength-building exercise  Relatedness for strength-building exercise  Enjoyment of strength-building exercise  Perceived intrinsic benefits of strength-building exercise (social, physical and emotional)  Enjoyment of the REACT programme (Intervention group)  Credibility /identification with the session facilitators (Intervention group only) |  | X  X  X  X  X  X  X  X  X  X  X | X  X  X  X  X  X  X  X  X  X  X  X  X  X  X  X  X | X  X  X  X  X  X  X  X  X  X  X  X  X  X  X  X  X | X  X  X  X  X  X  X  X  X  X  X  X |
| Qualitative Interviews |  |  | X | X | X |
| Focus groups |  |  |  | X | X |

**Table 1: Process measures taken at each time point**

**4.3 Analyses**

A detailed process evaluation analysis will be specified in due course. However, the types of analysis needed are either implied by or mentioned alongside the hypotheses above.

**5. Qualitative process evaluation**

**5.1 Research Questions**

The qualitative process evaluation will address five overarching questions:

RQ1. Was the intervention delivered as planned? Variations in intervention delivery by exercise specialists, including feedback on REACT training and implementation challenges will be investigated and recorded, as will variability in the acceptance/ receipt of the intervention by participants.

RQ2. Do any observed variations in delivery explain effectiveness / ineffectiveness of the intervention on physical function outcomes?

RQ3. What were the factors associated with engagement with REACT sessions? What made participants adhere to or drop-out from the programme? What made them volunteer or not for the Ambassadors programme?

RQ4. Do theorised mechanisms explain any observed impact on physical function and physical activity? Theorised change mechanisms, including key human needs (autonomy-relatedness-competence) identified in the Self Determination Theory, and other psychological and behaviour change processes (see section 2) will be investigated as mediators of intervention effects on physical function and physical activity.

RQ5. What other factors are associated with variation in intervention effectiveness among intervention recipients? Factors to be explored will include differences in participant characteristics (e.g. context/circumstances, ethnicity, deprivation index, beliefs and cognitions), perception of social connectedness and bonding within (and external to) groups, engagement with partner organisations, involvement with other activities offered by the same provider.

RQ6. What factors (including elements of the REACT intervention) help to support ongoing PA and exercise after the 12 months intervention period (i.e. between 12 and 24 months?

RQ7. What were participants’, facilitators’ and provider organisations’ experiences of the REACT Ambassadors programme? Variations in programme delivery by facilitators, and the activities engaged in by Ambassadors including perceived benefits or disbenefits and implementation challenges will be investigated and recorded.

RQ8. Why did REACT partners decide to continue (or not continue) delivering the REACT exercise programme after the completion of the 12 month intervention? In what ways did the REACT intervention help to support ongoing PA and exercise after the 12 months intervention period (i.e. between 12 and 24 months)

These questions will be addressed in four distinct studies, data from which will be analysed using both qualitative and quantitative methods that will, collectively, constitute the process evaluation.

**5.2 Study 1 Tracking the Experiences of Participants throughout the study: Repeated interviews addressing RQs 2,3,4,5, 7**

**Participants**: 20 participants (5 at each centre (Bath/Bristol/Exeter/Birmingham)).

**Data collection:** For intervention arm participants only, the initial *face-to-face meeting of participants with their exercise leader* (45-60 minutes) will, with permission, be audio recorded by the exercise leader. This will provide data about the participants’ initial expectations and motivations for taking part in the programme. Twenty participants will be purposively selected by members of the process evaluation team to represent a range of age, ethnicity, intervention response (SPPB change from 0 to 6 months), intervention adherence (number of sessions attended in the first 6 months) and will include men and women and participants at all three sites. Selection will be facilitated by review of baseline data as provided by CTU via the web-based database. Topic guides will be developed for the 6,12 and 24 month interviews. The interviews will be conducted by the PhD student and the research assistants at each site. Verbatim meeting and interview transcripts will be categorised and organised using computer software NVIVO.

The research team will, with permission, *interview* each of these 20 participants, preferably on their own, at 6 months after the baseline visit (i.e. after participants have completed the adoption phase and they have entered the maintenance phase of the intervention), 12 months (post-intervention) and 24 months (follow-up) after the baseline visit, and audio record these interviews. All recorded meetings and the three interviews will be recorded verbatim. The researchers will summarise the content of the interview at the end of the discussion and invite the participants to add anything else they would like to share. The interviewees will be asked if they would like a copy of the summarised findings. This will be sent through the postal system and the participants will be invited to add comments if they wish.

**Analysis:** For each participant, transcripts of one face to face meeting, and three interviews will be available for framework analysis. A researcher will listen to the audio recordings several times to familiarize herself with the data. Using NVIVO computer software, sections of data related to the aims will be assigned a code that summarizes the content either descriptively or interpretively. Codes with common features will be grouped together in predefined themes or new, emerging themes, before finally being assigned to interpretive overarching themes. Data about self-reported behaviour from the interviews will be compared with quantitative data on physical activity, exercise and session attendance collected during the study. Other members of the team will conduct independent analyses of subsets of the data, and the qualitative team will meet regularly to discuss their coding. Detailed notes of these discussions will be kept to help refine the analyses and to capture additional questions that could be answered from the data. Research reflexive memo notes will be used to assure transparency and trustworthiness of the analysis.

Participants’ observed and self-reported responses to the intervention and their link to overall use and perceived benefit, will be explored to identify interpersonal processes that shape effectiveness or ineffectiveness of the intervention. At 6 months, participants’ engagement with, response to the adoption phase of REACT (the structured exercise programme and the social/educational sessions) will be characterised and differences between participants noted. At 12 and 24 months overall evaluation of the intervention and maintenance of attendance and active lifestyle will be assessed and linked to responses at 6-months. This will allow a qualitative description of participants’ experiences, potential pathways and barriers to maintenance/involvement with other local initiatives. The analyses will be carried out by the lead qualitative researcher (RC) and AS with input from CG and JW, and the synthesis carried out by all four members of the qualitative team (RC,AS,CG,JW).

**5.3 Study 2 Investigation of Experiences of REACT exercise leaders and provider organisations addressing RQs 1,5.**

**Participants:** Up to 15 exercise leaders (at least 3 from each site) and all provider organisations at each site will be purposively sampled based on site of delivery.

**Data collection:** Focus groups will be conducted at 12 months from the time of intervention commencement in a mutually convenient venue. Focus groups are expected to last between 60-90 minutes. Focus groups will be conducted using a semi-structured interview guide allowing and encouraging participants to express their views. The researcher leading the exercise leaders’ and community providers’ interviews will work closely with the researcher conducting the participant interviews and review the topic guide throughout the study so that the questions are informed by relevant emerging topics. The research will also be guided by answers from the exercise leaders and the community providers and by further probing asking such as “tell me more about?” or “tell me how that made you feel?” Other techniques to enhance the interview include reflecting back on what was said, using non-verbal communication to show that the researcher is actively listing, for example, nodding, sitting forward, use of silence etc. The researcher will summarise the content of the interview at the end of the discussion and invite the participants to add anything else they would like to share. The interviewees will be asked if they would like a copy of the summarised findings. This will be sent through the postal system and the participants will be invited to add comments if they wish. The interviews will be carried out by the PhD student and the research assistants.

The researcher will write field notes at the end of each interview detailing how the interview was performed; reflect on their own performance and influence on the interview; how interviewees responded to the questions and initial thoughts about the main points arising from the interview.

All provider organisations will be assigned a code to ensure they remain anonymous. All other interviewees will have already been assigned a code. All focus groups will be audio recorded with the participants’ permission and the interviews will be stored on encrypted laptops and a secure data base at the University of Bath. In transcripts, all identifiable information will be removed. No participant will be identified in any publication. A thank you letter for participating will be sent to the participant after the interview and a summary of the findings will be provided in due course (if desired by the participant).

**Analysis:** The data from both the individual interviews and the focus groups audio-recordings will be transcribed verbatim either by an experienced transcriber/secretary or a specialist software. Data analyses will use similar methods as applied in study 1. The analysis will be conducted by the PhD student and the process evaluation research team.

**5.4 Study 3 Investigation of Experiences of REACT Ambassadors addressing RQ5**

**Participants**: Up to 30 REACT Ambassadors (up to 9 Ambassadors at each site) will be purposively sampled based on site of delivery.

**Data collection**: Three focus groups will be conducted at 24 months from the time of intervention commencement in a mutually convenient venue. Focus groups are expected to last between 60-90 minutes. Focus groups will be conducted using a semi-structured interview guide focusing on the suitability of the Ambassadors training, challenges in implementing the programme and level of success of the programme. The interviews will be carried out by the PhD student and the research assistants.

**Analysis:** The data from the focus groups audio-recordings will be transcribed verbatim either by an experienced transcriber/secretary or a specialist software. Data analyses will use similar methods as applied in study 1. The analysis will be conducted by the PhD student and the process evaluation research team.

**6. Assessment of intervention and training fidelity**

Fidelity of intervention delivery will be optimised and assessed using a range of the strategies outlined by the NIH Behaviour Change Consortium to assess and reinforce intervention fidelity^[8]^. To maximise and monitor trial fidelity we will: (i) optimise the “design fidelity” (consistency of intervention content and training with the underpinning theory and the intervention logic model) by reviewing the training and intervention materials ( KF, AS and CG) to ensure that all elements relate to the theoretical basis and logic model described above (ii) recruit REACT trainers with appropriate skills and experience, (iii) develop an accessible, standardised intervention manual, (iv) implement standardised REACT ‘trainer training’, (v) train more REACT trainers than needed to accommodate illness or withdrawal, and (vi) monitor delivery fidelity via recording of one-to-one consultation meetings for 20 participants and a sample of 4 sessions per intervention provider-pair (i.e. a minimum of 20 sessions) and the application of a ‘fidelity checklist’. This approach worked well in our NIHR-funded EARS study^[9]^ and our REACH-HF study^[10]^ (vii) check for and actively promote intervention “receipt” and “enactment” by including opportunities to check participant understanding of the correct performance of exercises and progress reviews to check enactment outside of the REACT sessions (especially following withdrawal of one session per week after 12 weeks and the other session at 12 months, with targeted planning activities around each transition).

**6.1 Study 4 Fidelity checks addressing RQ1**

A mixed methods assessment of intervention fidelity will include the following elements:

*Data collection*: A fidelity checklist, specifying intended intervention delivery techniques and processes, will be developed and piloted during the internal pilot study (April to September 2016). This will be applied to a purposive sample of 4 sessions per intervention provider-pair (i.e. a minimum of 20 sessions) and 20 individual face-to-face session recordings. The sampling of sessions will reflect diversity of delivery across providers (four sessions per provider-pair) and will aim to capture theoretically important content (e.g. sessions pre and post the transition to one session per week to capture crucial planning and problem-solving processes, the session on facilitation of identity change).

*Quantitative analysis:* Intervention fidelity scoring and analysis will be carried out by the PhD student and CG. An MSc (or other) student will act as a third coder and independently score a subgroup of 30 session recordings to help establish inter-rater reliability. Descriptive data (means and 95% confidence intervals) will be reported representing fidelity of delivery on each item of the checklist (each item is scored 0 to 6, using a Dreyfus competence-rating scale). The data will be summarised by facilitator-pairing, by site and overall (across all 4 sites).

*Qualitative analysis:* We will also generate a qualitative summary of the recorded sessions, highlighting examples of theorised (and non-theorised) intervention processes in practice, as well as examples of good and poor delivery practice. This analysis will help to interpret or contextualise the data on intervention adherence and effectiveness (e.g. explanations about why the intervention might work better for some people than others, barriers to engagement with the intervention).

Overall, the above analyses will clarify how well intervention components were delivered and received by participants and may identify components that were less well delivered. It will also allow researchers to describe variability in fidelity of delivery across sites and facilitators. Variability in the acceptance/ receipt of the intervention by participants might also be indicated by the number of exercise and social-education sessions attended.

References

1. Bandura A: *Social foundations of thought and action: A social cognitive theory.* Englewood Cliffs, NJ: Prentice-Hall; 1986.

2. Bandura A: **The primacy of self-regulation in health promotion.** *Applied Psychology-An International Review-Psychologie Appliquee-Revue Internationale* 2005, **54:**245-254.

3. Deci E, Ryan R: **Self-determination theory in health care and its relations to motivational interviewing: a few comments.** *International Journal of Behavioral Nutrition and Physical Activity* 2012, **9:**24.

4. Deci EL, Ryan RM: *Intrinsic motivation and self-determination in human behavior.* New York: Plenum Publishing Co; 1985.

5. Fortier M, Duda J, Guerin E, Teixeira P: **Promoting physical activity: development and testing of self-determination theory-based interventions.** *International Journal of Behavioral Nutrition and Physical Activity* 2012, **9:**20.

6. Harter S: *The construction of the self: A developmental perspective.* The Guilford Press; 1999.

7. Poltawski L, Greaves CJ, Briscoe S, Garside R: **Breaking bad (habits): A synthesis of qualitative research on weight loss maintenance.** *Health Psychology Reviews* 2015, **In submission**.

8. Bellg AJ, Borrelli B, Resnick B, Hecht J, Minicucci DS, Ory M, Ogedegbe G, Orwig D, Ernst D, Czajkowski S: **Enhancing treatment fidelity in health behavior change studies: Best practices and recommendations from the NIH behavior change consortium.** *Health Psychology* 2004, **23:**443-451.

9. Taylor AH, Thompson TP, Greaves CJ, Taylor RS, Green C, Warren FC, Kandiyali R, Aveyard P, Ayres R, Byng R, et al: **A pilot randomised trial to assess the methods and procedures for evaluating the clinical effectiveness and cost-effectiveness of Exercise Assisted Reduction then Stop (EARS) among disadvantaged smokers.** *Health Technology Assessment* 2014, **18**.

10. Taylor RS, Hayward C, Eyre V, Austin J, Davies R, Doherty P, Jolly K, Wingham J, Van Lingen R, Abraham C, et al: **The clinical effectiveness and cost-effectiveness of the Rehabilitation Enablement in Chronic Heart Failure (REACH-HF) facilitated self-care rehabilitation intervention in heart failure patients and caregivers: Rationale and protocol for a multicentre randomised controlled trial.** *BMJ Open* 2015, **In Press**.
