# Supplementary material for: A community-based physical activity intervention to prevent mobility-related disability for retired older people (REtirement in ACTion (REACT)): study protocol for a randomised controlled trial
Source: Trials. 2018 Apr 17;19:228. doi: 10.1186/s13063-018-2603-x (PMC5905123; doi:10.1186/s13063-018-2603-x)
Supplement: Supplementary file 4 — REACT Telephone Screening Form used to collect demographic data and undergo and initial eligibility assessment with potential participants. (DOCX 149 kb) [file 13063_2018_2603_MOESM4_ESM.docx]

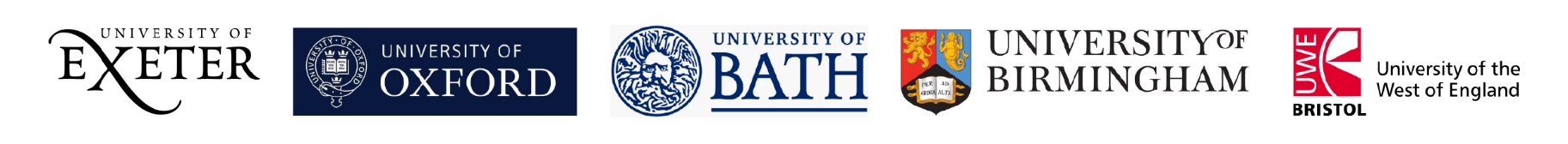
**REACT: Participant Screening**

| Date of Screening: | Researcher: | | | | | |
| --- | --- | --- | --- | --- | --- | --- |
| Participant ID: | | | Date of birth | | | |
| Verbal consent for telephone screening given | | | | | Yes | No |
| Gender | | | | | Male | Female |
| **GP Details**  GP Surname: GP 1^st^ Name: | | | | | | |
| Practice name: | | | | | | |
| Address: | | | | | | |
| Telephone number: | | | | | | |
| **If the participant doesn’t know all GP details (i.e. telephone number) complete these using an internet search after the telephone screening call. Some people will not see a specific GP in which case just record the GP practice details.** | | | | | | |
| How did you hear about the REACT study? | | | | | | |
| **Ethnicity**   1. White – British 2. White – Irish 3. Any other White background 4. Mixed – White and Black Caribbean 5. Mixed – White and Black African 6. Mixed – White and Asian 7. Any other Mixed background 8. Asian or Asian British – Indian 9. Asian or Asian British – Pakistani 10. Asian or Asian British – Bangladeshi 11. Any other Asian background 12. Black or Black British – Caribbean 13. Black or Black British – African 14. Any other Black background 15. Chinese 16. Any other ethnic group (please state) _______________ | | | | | (record number here) | |
| **What was your highest education level completed?**  1. Primary school  2. Middle school  3. Some secondary school  4. Completed secondary school  5. Some college or vocational training  6. Completed college or university  7. Completed graduate degree, or higher | | | | | (record number here) | |
| *“Now I’m going to run through a series of questions and when we get to the end I’ll explain whether you could be eligible to take part in REACT and if you are we’ll go onto a few more questions.”* | | | | | Yes | No |
| 1. Are you in full time work? | | | | | Yes | No |
| 1. A) Do you use a wheelchair? | | | | | Yes | No |
| B) Do you use a Zimmer frame? | | | | | Yes | No |
| 3. How would you find walking across a room? (With a walking stick is ok if needs a zimmer tick Unable) | | Easy  ⏷ | | A little difficult | Very difficult | Unable |
| 4. How easy would you find getting out of a low chair? | | Easy  ⏷ | | A little difficult | Very difficult | Unable |
| 1. If response is Easy ask: Would you normally use your hands to help you get up from the chair? | | Yes/No | | | | |
| 5. How easy would you find walking up a flight of stairs with no handrail or wall to lean on? | | Easy  ⏷ | | A little difficult | Very difficult | Unable |
| 6. How easy do you find walking on an uneven pavement without losing your balance? | | Easy  ⏷ | | A little difficult | Very difficult | Unable |
| **Does participant find all four of these easy and answered No to 4a*?*** | | | | | Yes | No |
| 7. Has a doctor ever advised you not to exercise?  **If yes**, Why was that?  (See researcher notes for advice depending on answer) | | | | | Yes | No |
| 1. Are you planning to move out of the area within the next 2 years? | | | | | Yes | No |
| 1. Is anyone else in your household taking part in REACT? | | | | | Yes | No |
| 1. Do you live in a residential care home or a nursing home? | | | | | Yes | No |

| 1. Have you been diagnosed by your GP with any of the following medical conditions: | | |
| --- | --- | --- |
| 1. Arthritis (either osteo or rheumatoid) that is so severe that it would prevent you from walking about 100 yards/metres, that’s about the length of a football pitch | Yes | No |
| 1. Parkinson’s disease | Yes | No |
| 1. Dementia | Yes | No |
| 1. Lung disease that requires using oral steroids (tablets) or supplemental oxygen? (Does not include using an inhaler) | Yes | No |
| 1. Severe kidney disease that requires dialysis | Yes | No |
| 1. Chest pain when walking one or two hundred yards or up a flight of stairs | Yes | No |
| 1. Do you have an implanted cardiac defibrillator? | Yes | No |
| 1. Have you ever had a cardiac arrest which required resuscitation? | Yes | No |
| 1. Within the last six months have you had major heart surgery, including valve replacement or bypass surgery? | Yes | No |
| 1. Do you have any other heart condition? **If yes** please specify:   *_____________________________________________________* | Yes | No |
| 1. Are you currently receiving radiation treatment and/or chemotherapy for cancer *Interviewer Note: Tamoxifen for breast cancer or hormonal therapy for any cancers is not chemotherapy* | Yes | No |
| 1. Are you awaiting knee or hip surgery? | Yes | No |
| 1. Within the last six months have you had spinal surgery? | Yes | No |
| 1. Have you been diagnosed with a terminal illness | Yes | No |

| 1. Are you planning to go into hospital any time in the next year for anything other than hip or knee surgery? | | Yes | | No |
| --- | --- | --- | --- | --- |
| 1. In a typical or normal week during the past 4 weeks, did you… | | | | |
| 1. Spend at least 20 minutes a week getting regular exercise?   Exercise includes activities like: brisk walking, jogging, weight lifting, cycling, aerobics, or dancing. | Yes | No | |  |
| 1. Dance? (such as square, folk, line, ballroom) (*Interviewer Note: Do not count aerobic dance)* | Yes | No | | ­­­_____  mins |
| 1. Walk uphill or hike uphill? | Yes | No | | _____  Mins |
| 1. Walk fast or briskly for exercise? | Yes | No | | _____  mins |
| 1. Do water exercises? (*Interviewer Note: Include only aqua aerobics exercise, not other swimming)* | Yes | No | | _____  Mins |
| 1. *Sum of minutes per week (items b,c,d,e)* | _________mins | | | |
| **Do any of the participant’s answers fall into any of the shaded boxes, or they find all three activities easy?** | Yes  ⏷ | | No | |
| **If ‘yes’, explain that:**  *“Unfortunately we can’t include you in the REACT programme at the moment (use the explanations in the Screening protocol), but thank you very much for your time. We will send you some information about Health Ageing that you might find useful.* | | | | |
| **If being unable to walk across a room without assistance is due to a temporary condition record the temporary condition and treat as a temporary exclusion.**  **Condition:** _______________________________________________  **Date to call back:** _________________________________________ | | | | |
| **If no answers fall into the shaded boxes explain that:**  *“You could be eligible to take part in REACT, but I will just ask you a few more questions”* | | | | |

| 1. **Confidence in ability to get to sessions:**   *“None of us can predict exactly what will happen over the next year, holidays, illnesses and minor operations may crop up unexpectedly, but in general if you were allocated to the physical activity sessions how confident do you feel that you would usually be able to attend a REACT session at (insert venue) twice a week (insert days of the week if available) for the next three months and once a week for the following nine months.”* | | | | |
| --- | --- | --- | --- | --- |
| Quite confident | Not sure | | Not at all confident | |
| If not at all confident ask and record the reason(s) why:  ___________________________________________________________________  **If not confident because of the location of the venue or days of the week explain:** *“Other venues/days will become available later this year, which may be more convenient for you. Would you like us to call you back then? This will be around September time. (Note date below)*  **If participant lives relatively close to the venue but is not confident because of transport problems:** *“We could offer to help arrange transport for you. Would that make you more confident about attending?” (Indicate below if yes)*  **If still not confident, suggest that:** *“Would it be better if we called you back in a few months to see if you are feeling more capable of attending sessions?”* | | | | |
| Agreed to be called back  Date: _________________ | | Need transport to be arranged | | Preferred not to be called back |

**Temporary exclusions**

| *“I will just continue to ask a few more questions…”* |  | |  | |
| --- | --- | --- | --- | --- |
| 1. Within the last 6 months have you had a hip fracture? | Yes | | No | |
| 1. Within the last 6 months have you had a hip or knee replacement? | Yes | | No | |
| 1. Within the last 6 months have you had a heart attack (or “myocardial infarction”) that required overnight hospitalisation? | Yes | | No | |
| 1. Within the last 6 months have you had a stroke (does not include a transient ischemic attack (TIA) or mini-stroke)?   Did this result in any movement related impairment?  _______________________________________________________ | Yes | | No | |
| 1. Are you currently receiving physical therapy on your legs or to help with movement in your legs? | Yes | | No | |
| 1. Are you currently enrolled in another physical activity research or intervention study? **If yes:**   Study name: ______________________________________  Study end date:____________________________________ | Yes | | No | |
| **If participant answers fall into any of the shaded boxes, explain that:** *“We can’t include you in the REACT trial at the moment, but if you are happy for us to do so, we will contact you again in a few months to re-assess the situation.”* | | | | |
| **If ‘yes’:** *“Could you suggest a good date to call you back?”* Date:_________________ | | | | |
| **If ‘no’:** *“That is not a problem. Would you like us to post you an information pack which contains details of local activities and places where you will able to get health advice?”* | | Yes | | No |
| **If *none* of the responses fall into shaded boxes:** *“Just a few last questions…”* | | Yes | | No |
| 1. Is there a health reason not mentioned why you would be concerned about starting to be involved in more activities, or being a bit more active?   **If ‘yes’** ask for more details:  _______________________________________________________ | | Yes | | No |
| 1. Do you drive? | | Yes | | No |
| **If ‘yes’:** Do you have access to a car that you can use regularly? | | Yes | | No |
| **If ‘no’:** Do do you have access to other forms of transport? | | Yes | | No |

*“Thank you. That’s all I have to ask you at the moment, is there anything you’d like to ask me?”............................. “From what you have told me so far you could be eligible to take part in REACT so what we would like to do now is invite you to attend a session at (venue) so that we can meet face to face and conduct a few more simple screening tests such as asking you to walk 4 metres and answer a few more questions.”*

| 1. **Are you still happy to attend?** | Yes | No |
| --- | --- | --- |
| **Is this venue convenient for you?** | Yes | No |
| **If ‘No’: “***We will be running REACT in other areas around the city later this year. Would you prefer to be put on the waiting list for these other sessions?* | Yes | No |
| **If ‘Yes’, set date of call:** ___________________________________ | | |
| **If ‘No’ explain that: “***It may be possible to help you arrange transport to get to the sessions, does that make this venue more convenient?* | Yes | No |
| **If still ‘No’:** *‘That is not a problem at all, if you change your mind, please do get back in touch with us vial telephone, post or email.* | | |

**Explain the time and venue details of the screening session and discuss any transport requirements, explain travel expenses will be paid.**

*“It’s been nice talking to you. I’ll send you an email or post you a letter to confirm the details of the face-to-face screening session. Which would you prefer?”* ***POST / EMAIL***

*“OK, that’s it for today. If there are any more questions you want to ask you can call me anytime. Thank you very much for your time.”*

--------------------------------------------------------------------------------------------

*Researcher:-*

□ Record date details of measurement session sent _____________________

□ Finalise transport arrangements. Transport details _____________________

□ File Screening form

□ Enter details onto database Date ____________________
