# Supplementary material for: A community-based physical activity intervention to prevent mobility-related disability for retired older people (REtirement in ACTion (REACT)): study protocol for a randomised controlled trial
Source: Trials. 2018 Apr 17;19:228. doi: 10.1186/s13063-018-2603-x (PMC5905123; doi:10.1186/s13063-018-2603-x)
Supplement: Supplementary file 6 — REACT Case Report Form including all measures and items used to collect outcome data as described in Table 2. (DOC 1427 kb) [file 13063_2018_2603_MOESM6_ESM.doc]

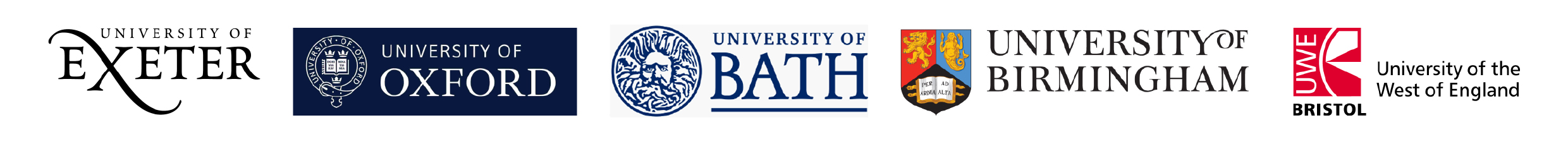


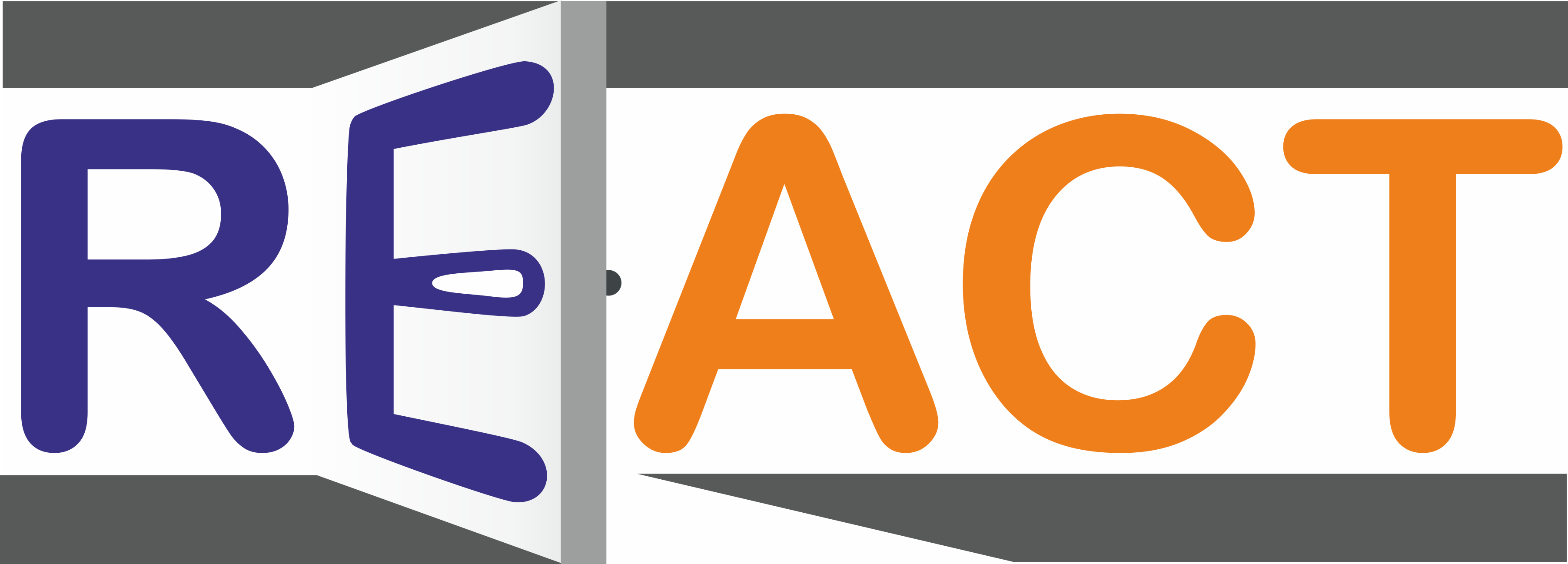


(REtirement in ACTion)

Questionnaire (V2)

We need your help to make our study a success.

Remember that…

- **we want to know what you think about things, and understand more about your feelings and opinions**
- **there are no right or wrong answers – please just be as honest as you feel you can be**
- **we suggest you go with your ‘gut reaction’ and don’t over-think answers**
- **everything you tell us will be kept strictly confidential (secret) and you’re free to decline answering any questions you’re not comfortable with**

**For Researcher Use**

**Measures 1 ❒ Measures 2 ❒ Measures 3 ❒ Measures 4 ❒**

**Date: _____________________ Researcher initials: _________**

**Was another person present/contributing during the questionnaire delivery?**

**Yes/No If ‘yes,’ who: _____________________**

**Data entry researcher**

**ID CODE: ___________________ Data entered: __________ Initials: _______**

**
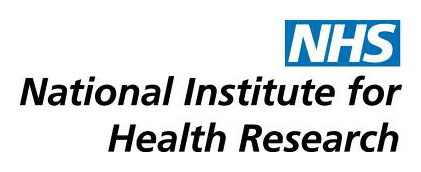
**

**SCREENING SECTION**

**A. SPPB Physical function tests**

**4m walking speed ( ) – Strike through following table if not used**

| **ATTEMPT** | **Time (circle best of 2)** | **≤4.81s** | **4.82-6.20s** | **6.21-8.70s** | **8.71-59.99s** | **>60s/Unable** | **Aid used? (circle)** | **Code non-attempt/fail** | **SCORE** |
| --- | --- | --- | --- | --- | --- | --- | --- | --- | --- |
| **#1** |  | ❒ [4] | ❒ [3] | ❒ [2] | ❒ [1] | ❒ [0] | No / Stick / Oth |  |  |
| **#2** |  | ❒ [4] | ❒ [3] | ❒ [2] | ❒ [1] | ❒ [0] | No / Stick / Oth |  |

BASELINE ONLY (If participant does not complete the 4 metre walk they are not eligible to participate in REACT. Do not continue the tests. Explain the situation. Thank them for their time. Offer refreshments. Spend some time explaining the information pack and ensuring their transport back home is available).

| **Standing balance (🗹 )** | **Side-by-side** | | **Semi-tandem** | | **Full tandem stand** | | **BALANCE SCORE (SUM)** |
| --- | --- | --- | --- | --- | --- | --- | --- |
| **Time** (record *only if* <10s) |  | |  | |  | |  |
| **Code non-attempt /fail [0]** |  | |  | |  | |  |
|  | **< 10 s** | ❒ [0] END | < 10 s | ❒ [0] END | < 3 s | ❒ [0] |  |
|  | **≥ 10 s** | ❒ [1] | ≥ 10 s | ❒ [1] | 3-9.99 s | ❒ [1] |  |
|  |  | |  |  | ≥ 10 s | ❒ [2] |  |

**Chair rise(s) (** )

| **Completed single chair stand? (circle)** | **Time** | **≤11.19s** | **11.20–13.69s** | **13.70-16.69s** | **16.70-59.99s** | **>60s/Unable to complete 5 stands/used hands** | **Code non-attempt/fail** | **SCORE** |
| --- | --- | --- | --- | --- | --- | --- | --- | --- |
| **Yes / No / Used hands** [0] |  | ❒ [4] | ❒ [3] | ❒ [2] | ❒ [1] | ❒ [0] |  |  |

**3m walking speed ( )** – Strike through following table if not used

| **ATTEMPT** | **Time (circle best of 2)** | **≤3.61s** | **3.62-4.65s** | **4.66-6.52s** | **6.53-59.99s** | **>60s/ Unable** | **Aid used? (circle)** | **Code non-attempt/fail** | **SCORE** |
| --- | --- | --- | --- | --- | --- | --- | --- | --- | --- |
| #1 |  | ❒ [4] | ❒ [3] | ❒ [2] | ❒ [1] | ❒ [0] | No / Stick / Oth |  |  |
| #2 |  | ❒ [4] | ❒ [3] | ❒ [2] | ❒ [1] | ❒ [0] | No / Stick / Oth |  |

| **CODES for non-attempt or failure**  **1** Tried but unable  **2** Participant could not hold position/stand/walk unassisted  **3** Not attempted, researcher felt unsafe  **4** Not attempted, participant felt unsafe  **5** Participant unable to understand instructions  **6** Other (specify in tables above)  **7** Participant refused |  | **PARTICIPANT’S FINAL SCORE** |  |
| --- | --- | --- | --- |

BASELINE ONLY If participant scores lower than 4 or more than 9 they are not eligible to participate in REACT (a score of 4-9 is required to participate) do not continue the tests. Explain the situation. Thank them for their time. Offer refreshments. Spend some time explaining the information pack and ensuring their transport home is available.

**Baseline Measurement Section**

**B. Montreal Cognitive Assessment (MoCA)**


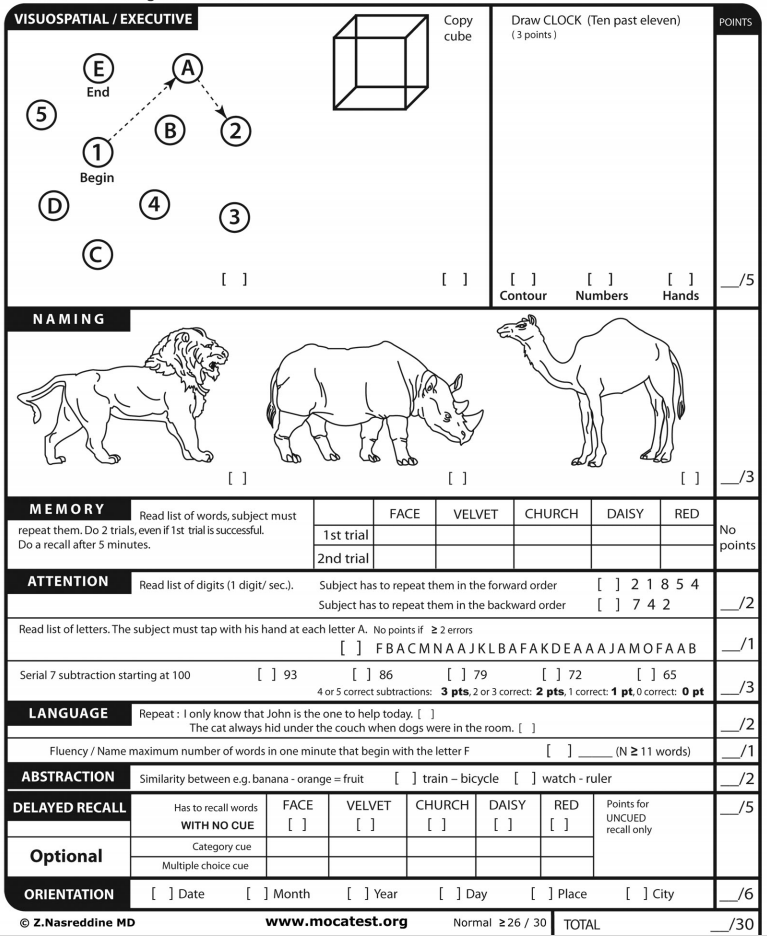


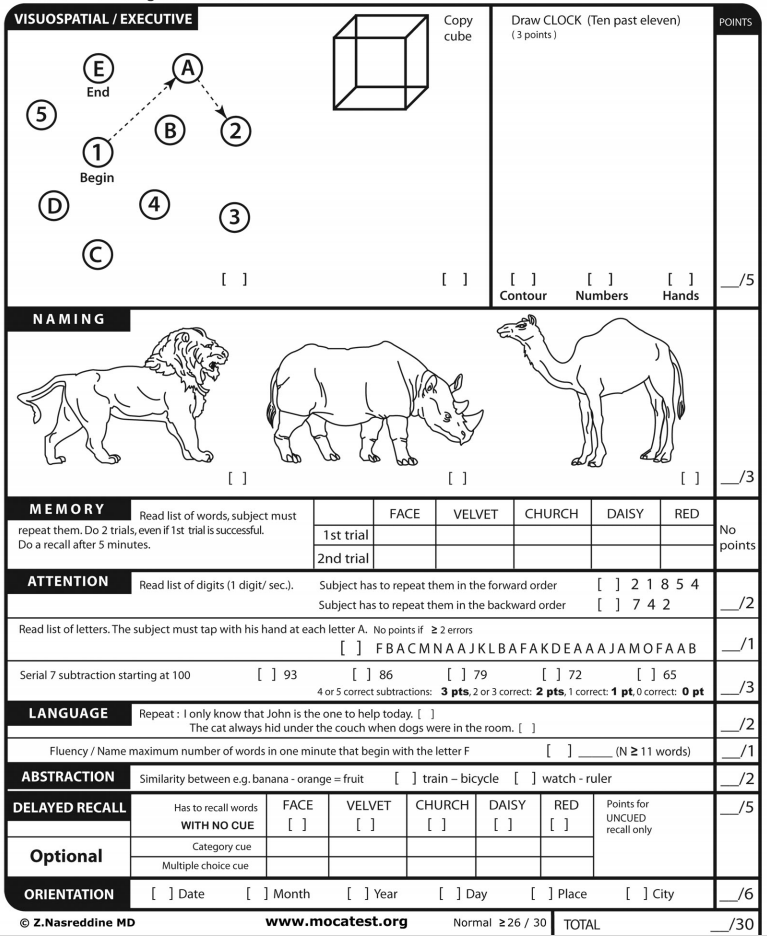


Score as instructed in the scoring sheet. If score is less than 10 we may need to contact the participant’s GP to ask if they should take part. Continue with measures but on completion discuss with the measures session leader.

**C. Everyday activities**

**INSTRUCTIONS:**

Please complete this questionnaire by either circling the correct response

or filling in the blank. Here is an example:

During the past 7 days, how often have you seen the sun?

| [0.] NEVER | [1.] SELDOM  (1-2 DAYS) | [2.] SOMETIMES  (3-4 DAYS) | [3.] OFTEN  (5-7 DAYS) |
| --- | --- | --- | --- |

Answer all items as accurately as possible. All information is strictly confidential.

**C. Everyday activities**

**INSTRUCTIONS:**

Please complete this questionnaire by either circling the correct response. Here is an example:

During the past 7 days, how often have you seen the sun?

| [0.] NEVER | [1.] SELDOM  (1-2 DAYS) | [2.] SOMETIMES  (3-4 DAYS) | [3.] OFTEN  (5-7 DAYS) |
| --- | --- | --- | --- |

Answer all items as accurately as possible. All information is strictly confidential.

**LEISURE TIME ACTIVITY**

1. Over the past 7 days, how often did you participate in sitting activities such as reading, watching TV or doing handcrafts?

| [0.] NEVER  ⏷  GO TO Q#2 | [1.] SELDOM  (1-2 DAYS)  ⏷ | [2.] SOMETIMES  (3-4 DAYS)  ⏷ | | [3.] OFTEN  (5-7 DAYS)  ⏷ |
| --- | --- | --- | --- | --- |
|  | 1a. What were these activities?  __________________________________________________  __________________________________________________  1b. On average, how many hours per day did you engage in these sitting activities? | | | |
|  | [1.] LESS THAN 1 HOUR | | [2.] 1 BUT LESS THAN 2 HOURS | |
|  | [3.] 2-4 HOURS | | [4.] MORE THAN 4 HOURS | |

2. Over the past 7 days, how often did you walk outside your home or yard for any reason? For example, for fun or exercise, walking to work, walking the dog, etc.?

| [0.] NEVER  ⏷  GO TO Q#3 | [1.] SELDOM  (1-2 DAYS)  ⏷ | [2.] SOMETIMES  (3-4 DAYS)  ⏷ | | [3.] OFTEN  (5-7 DAYS)  ⏷ |
| --- | --- | --- | --- | --- |
|  | 2a. On average, how many hours per day did you spend walking? | | | |
|  | [1.] LESS THAN 1 HOUR | | [2.] 1 BUT LESS THAN 2 HOURS | |
|  | [3.] 2-4 HOURS | | [4.] MORE THAN 4 HOURS | |

3. Over the past 7 days, how often did you engage in light sport or recreational activities such as bowling, golf with a cart, shuffleboard, fishing from a boat or pier or other similar activities?

| [0.] NEVER  ⏷  GO TO Q#4 | [1.] SELDOM  (1-2 DAYS)  ⏷ | [2.] SOMETIMES  (3-4 DAYS)  ⏷ | | [3.] OFTEN  (5-7 DAYS)  ⏷ |
| --- | --- | --- | --- | --- |
|  | 3a. What were these activities?  __________________________________________________  __________________________________________________  3b. On average, how many hours per day did you engage in these light sport or recreational activities? | | | |
|  | [1.] LESS THAN 1 HOUR | | [2.] 1 BUT LESS THAN 2 HOURS | |
|  | [3.] 2-4 HOURS | | [4.] MORE THAN 4 HOURS | |

4. Over the past 7 days, how often did you engage in moderate sport and recreational activities such as doubles tennis, ballroom dancing, hunting, ice skating, golf without a cart, softball or other similar activities?

| [0.] NEVER  ⏷  GO TO Q#5 | [1.] SELDOM  (1-2 DAYS)  ⏷ | [2.] SOMETIMES  (3-4 DAYS)  ⏷ | | [3.] OFTEN  (5-7 DAYS)  ⏷ |
| --- | --- | --- | --- | --- |
|  | 4a. What were these activities?  __________________________________________________  __________________________________________________  4b. On average, how many hours per day did you engage in these moderate sport and recreational activities? | | | |
|  | [1.] LESS THAN 1 HOUR | | [2.] 1 BUT LESS THAN 2 HOURS | |
|  | [3.] 2-4 HOURS | | [4.] MORE THAN 4 HOURS | |

5. Over the past 7 days, how often did you engage in strenuous sport and recreational activities such as jogging, swimming, cycling, singles tennis, aerobic dance, skiing (downhill or cross-country) or other similar activities?

| [0.] NEVER  ⏷  GO TO Q#6 | [1.] SELDOM  (1-2 DAYS)  ⏷ | [2.] SOMETIMES  (3-4 DAYS)  ⏷ | | [3.] OFTEN  (5-7 DAYS)  ⏷ |
| --- | --- | --- | --- | --- |
|  | 5a. What were these activities?  __________________________________________________  __________________________________________________  5b. On average, how many hours per day did you engage in these strenuous sport and recreational activities? | | | |
|  | [1.] LESS THAN 1 HOUR | | [2.] 1 BUT LESS THAN 2 HOURS | |
|  | [3.] 2-4 HOURS | | [4.] MORE THAN 4 HOURS | |

6. Over the past 7 days, how often did you do any exercises specifically to increase muscle strength and endurance, such as lifting weights or pushups, etc.?

| [0.] NEVER  ⏷  GO TO Q#7 | [1.] SELDOM  (1-2 DAYS)  ⏷ | [2.] SOMETIMES  (3-4 DAYS)  ⏷ | | [3.] OFTEN  (5-7 DAYS)  ⏷ |
| --- | --- | --- | --- | --- |
|  | 6a. What were these activities?  __________________________________________________  __________________________________________________  6b. On average, how many hours per day did you engage in exercises to increase muscle strength and endurance? | | | |
|  | [1.] LESS THAN 1 HOUR | | [2.] 1 BUT LESS THAN 2 HOURS | |
|  | [3.] 2-4 HOURS | | [4.] MORE THAN 4 HOURS | |

**HOUSEHOLD ACTIVITY**

Please circle your answer

7. During the past 7 days, have you done any light housework, such as dusting or washing dishes?

[1.] NO [2.] YES

8. During the past 7 days, have you done any heavy housework or chores, such as vacuuming, scrubbing floors, washing windows, or carrying wood?

[1.] NO [2.] YES

9. During the past 7 days, did you engage in any of the following activities?

Please answer YES or NO for each item by circling your response.

|  |  | NO | YES |
| --- | --- | --- | --- |
| a. | Home repairs like painting, wallpapering, electrical work, etc. | 1 | 2 |
| b. | Lawn work or yard care, including snow or lead removal, wood chopping etc. | 1 | 2 |
| c. | Outdoor gardening | 1 | 2 |
| d. | Caring for another person, such as children, dependent spouse, or an other adult | 1 | 2 |

**WORK-RELATED ACTIVITY**

Please circle your answer

10. During the past 7 days, did you work for pay or as a volunteer?

[1.] NO [2.] YES

If **NO** please go to **SECTION D**, if **YES** please give details below:

| 10a. How many hours per week did you work for pay and/or as a volunteer?  _____________________________ HOURS |
| --- |
| 10b. Which of the following categories best describes the amount of physical activity required on your job and/or volunteer work? Circle one number. |
| [1] Mainly sitting with slight arm movements.  [**Examples:** office worker, watchmaker, seated assembly line worker, bus driver, etc.] |
| [2] Sitting or standing with some walking.  [**Examples:** cashier, general office worker, light tool and machinery worker.] |
| [3] Walking, with some handling of materials generally weighing less than 40 pounds.  [**Examples:** mailman, waiter/waitress, construction worker, heavy tool and machinery worker.] |
| [4] Walking and heavy manual work often requiring handling of materials weighing over 50 pounds.  [**Examples:** lumberjack, stone mason, farm or general labourer]. |

**D. Ageing Well Profile**

In this section, we’re interested in your well-being during the **last month.** Choose the box (α) that best describes your feelings and thoughts about your health.

| **About your life…** |  | | | | |
| --- | --- | --- | --- | --- | --- |
| **During the last month:** | **Not really Sort of Really**  **true true true**  **for me for me for me** | | | | |
| I have managed to sort out all my needs by myself. | 1  ⬜ | 2  ⬜ | 3  ⬜ | 4  ⬜ | 5  ⬜ |
| In everyday life, I have not needed to rely on others. | 1  ⬜ | 2  ⬜ | 3  ⬜ | 4  ⬜ | 5  ⬜ |
| I have been able to take good care of myself. | 1  ⬜ | 2  ⬜ | 3  ⬜ | 4  ⬜ | 5  ⬜ |
| My life has given me a sense of accomplishment. | 1  ⬜ | 2  ⬜ | 3  ⬜ | 4  ⬜ | 5  ⬜ |
| I have felt some good changes in myself. | 1  ⬜ | 2  ⬜ | 3  ⬜ | 4  ⬜ | 5  ⬜ |
| My life has been really interesting. | 1  ⬜ | 2  ⬜ | 3  ⬜ | 4  ⬜ | 5  ⬜ |
| I have felt that I have improved myself. | 1  ⬜ | 2  ⬜ | 3  ⬜ | 4  ⬜ | 5  ⬜ |

| **About your health and fitness…** |  | | | | |
| --- | --- | --- | --- | --- | --- |
| **During the last month:** | **Not really Sort of Really**  **true true true**  **for me for me for me** | | | | |
| I have usually woken up fresh and rested | 1  ⬜ | 2  ⬜ | 3  ⬜ | 4  ⬜ | 5  ⬜ |
| My body has felt strong enough to do what I wanted to do | 1  ⬜ | 2  ⬜ | 3  ⬜ | 4  ⬜ | 5  ⬜ |
| On a day to day basis I have felt lively and healthy | 1  ⬜ | 2  ⬜ | 3  ⬜ | 4  ⬜ | 5  ⬜ |
| I have not had many pains or much discomfort | 1  ⬜ | 2  ⬜ | 3  ⬜ | 4  ⬜ | 5  ⬜ |
| My body has felt old and has limited what I could do | 1  ⬜ | 2  ⬜ | 3  ⬜ | 4  ⬜ | 5  ⬜ |
| I have been able to keep going for longer than most people my age | 1  ⬜ | 2  ⬜ | 3  ⬜ | 4  ⬜ | 5  ⬜ |

**** Note for researcher: check correct completion of highlighted item***

| **About your feelings…** |  | | | | |
| --- | --- | --- | --- | --- | --- |
| **During the last month:** | **Not really Sort of Really**  **true true true**  **for me for me for me** | | | | |
| I have had no doubts about who I am or my worth | 1  ⬜ | 2  ⬜ | 3  ⬜ | 4  ⬜ | 5  ⬜ |
| I have rarely felt confused | 1  ⬜ | 2  ⬜ | 3  ⬜ | 4  ⬜ | 5  ⬜ |
| I have not worried much about my life | 1  ⬜ | 2  ⬜ | 3  ⬜ | 4  ⬜ | 5  ⬜ |
| I have been able to concentrate well when I wanted | 1  ⬜ | 2  ⬜ | 3  ⬜ | 4  ⬜ | 5  ⬜ |
| I have had complete confidence in myself and my decisions | 1  ⬜ | 2  ⬜ | 3  ⬜ | 4  ⬜ | 5  ⬜ |
| I have felt contented and happy with myself | 1  ⬜ | 2  ⬜ | 3  ⬜ | 4  ⬜ | 5  ⬜ |
| I have been in a good mood more often than not | 1  ⬜ | 2  ⬜ | 3  ⬜ | 4  ⬜ | 5  ⬜ |

| **About your social life…** |  | | | | |
| --- | --- | --- | --- | --- | --- |
| **During the last month:** | **Not really Sort of Really**  **true true true**  **for me for me for me** | | | | |
| I have had plenty of people available to share my problems with | 1  ⬜ | 2  ⬜ | 3  ⬜ | 4  ⬜ | 5  ⬜ |
| I have felt that people have enjoyed my company | 1  ⬜ | 2  ⬜ | 3  ⬜ | 4  ⬜ | 5  ⬜ |
| I have rarely felt lonely or isolated | 1  ⬜ | 2  ⬜ | 3  ⬜ | 4  ⬜ | 5  ⬜ |
| I have had lots of friends who I wanted to spend time with | 1  ⬜ | 2  ⬜ | 3  ⬜ | 4  ⬜ | 5  ⬜ |
| I have spent a lot of my time with friends and acquaintances | 1  ⬜ | 2  ⬜ | 3  ⬜ | 4  ⬜ | 5  ⬜ |
| My social life has been as good, as I would have liked | 1  ⬜ | 2  ⬜ | 3  ⬜ | 4  ⬜ | 5  ⬜ |
| I have been able to give support and friendship to other people | 1  ⬜ | 2  ⬜ | 3  ⬜ | 4  ⬜ | 5  ⬜ |

**E. The Sleep Condition Indicator**

Please circle your answer

|  |  |  | **Score** |  |  |
| --- | --- | --- | --- | --- | --- |
| **Item** | **4** | **3** | **2** | **1** | **0** |
| **Thinking about a typical night in the last month…** | | |  |  |  |
| 1. … how long does it take you to fall asleep? | 0-15 min | 16-30 min | 31-45 min | 46-60 min | ≥61 min |
| 2. …if you then wake up during the night … how long are you awake for in total? (add all the wakenings up) | 0-15 min | 16-30 min | 31-45 min | 46-60 min | ≥61 min |
| 3. …how many nights a week do you have a problem with your sleep? | 0-1 | 2 | 3 | 4 | 5-7 |
| 4. … how would you rate your sleep quality? | Very good | Good | Average | Poor | Very poor |
| **Thinking about the past month, to what extent has poor sleep…** | | | |  |  |
| 5. …affected your mood, energy, or relationships? | Not at all | A little | Somewhat | Much | Very much |
| 6. …affected your concentration, productivity, or ability to stay awake? | Not at all | A little | Somewhat | Much | Very much |
| 7. …troubled you in general? | Not at all | A little | Somewhat | Much | Very much |
| **Finally …** |  |  |  |  |  |
| 8. …how long have you had a problem with your sleep? | I don’t have a problem/  Less than 1 month | 1-2  months | 3-6 months | 7-12 months | More than 1 year |

**F. Life Satisfaction**

Please circle your answer

1. Overall, how satisfied are you with your life nowadays?

| Not at all | | Completely | |
| --- | --- | --- | --- |
| 0 | 1 2 3 4 5 6 7 8 9 | | 10 |

**G. SF-36**

1. In general, would you say your health is:

| Excellent | Very good | Good | Fair | Poor |
| --- | --- | --- | --- | --- |
| 1 | 2 | 3 | 4 | 5 |

2. Compared to one year ago, how would you rate your health in general now:

| Much better | Somewhat better | About the same | Somewhat worse | Much worse |
| --- | --- | --- | --- | --- |
| 1 | 2 | 3 | 4 | 5 |

The following items are about activities you might do during a typical day. Does **your health now limit you** in these activities? If so, how much?

(Circle One Number on Each Line)

|  | Yes, limited a lot | Yes,  limited a little | No,  not limited at all |
| --- | --- | --- | --- |
| 3. **Vigorous activities**, such as running, lifting heavy objects, participating in strenuous sports | [1] | [2] | [3] |
| 4. **Moderate activities**, such as moving a table, pushing a vaccuum cleaner, bowling, or playing golf | [1] | [2] | [3] |
| 5. Lifting or carrying groceries | [1] | [2] | [3] |
| 6. Climbing **several** flights or stairs | [1] | [2] | [3] |
| 7. Climbing **one** flight of stairs | [1] | [2] | [3] |
| 8. Bending, kneeling, or stooping | [1] | [2] | [3] |
| 9. Walking **more than a mile** | [1] | [2] | [3] |
| 10. Walking **several hundred yards** | [1] | [2] | [3] |
| 11. Walking **one hundred yards** | [1] | [2] | [3] |
| 12. Bathing or dressing yourself | [1] | [2] | [3] |

During the **past 4 weeks**, have you had any of the following problems with your work or other regular daily activities **as a result of your physical health?**

(Circle One Number on Each Line)

|  | Yes | No |
| --- | --- | --- |
| 13. Cut down the amount of time you spent on work or other activities | 1 | 2 |
| 14. **Acomplished less** than you would like | 1 | 2 |
| 15. Were limited in the **kind** of work or other activities | 1 | 2 |
| 16. Had **difficulty** performing the work or other activities  (for example, it took extra effort) | 1 | 2 |

During the past **4 weeks**, have you had any of the following problems with your work or other regular daily acivities **as a result of any emotional problems** (such as feeling depressed or anxious)?

(Circle One Number on Each Line)

|  | Yes | No |
| --- | --- | --- |
| 17. Cut down the **amount of time** you spent on work or  other activities | 1 | 2 |
| 18. **Accomplished less** than you would like | 1 | 2 |
| 19. Didn’t do work or other activities as **carefully** as usual | 1 | 2 |

20. During the **past 4 weeks**, to what extent has your physical health or emotional problems interferred with your normal social activities with family, friends, neighbours or groups?

(Circle One Number)

| Not at all | Slightly | Moderately | Quite a bit | Extremely |
| --- | --- | --- | --- | --- |
| 1 | 2 | 3 | 4 | 5 |

21. During the **past 4 weeks**, how much bodily pain have you had?

(Circle One Number)

| None | Very mild | Mild | Moderate | Severe | Very severe |
| --- | --- | --- | --- | --- | --- |
| 1 | 2 | 3 | 4 | 5 | 6 |

22. During the **past 4 weeks**, how much did pain interfere with your normal work (including work both outside the home, and housework?)

(Circle One Number)

| Not at all | A little bit | Moderately | Quite a bit | Extremely |
| --- | --- | --- | --- | --- |
| 1 | 2 | 3 | 4 | 5 |

These questions are about how you feel and how things have been with you **during the past 4 weeks**. For each question, please give the one answer that comes closets to the way you have been feeling.

How much of the time during the **past 4 weeks** . . .

(Circle One Number on Each Line)

|  | All of the time | Most of the time | A good bit of the time | Some of the time | A little of the time | None of the time |
| --- | --- | --- | --- | --- | --- | --- |
| 23. Did you feel full of life? | 1 | 2 | 3 | 4 | 5 | 6 |
| 24. Have you been nervous? | 1 | 2 | 3 | 4 | 5 | 6 |
| 25. Have you felt so down in the dumps that nothing could cheer you up? | 1 | 2 | 3 | 4 | 5 | 6 |
| 26. Have you felt calm and peaceful? | 1 | 2 | 3 | 4 | 5 | 6 |
| 27. Did you have a lot of energy? | 1 | 2 | 3 | 4 | 5 | 6 |
| 28. Have you felt downhearted and low? | 1 | 2 | 3 | 4 | 5 | 6 |
| 29. Did you feel worn out? | 1 | 2 | 3 | 4 | 5 | 6 |
| 30. Have you been a happy person? | 1 | 2 | 3 | 4 | 5 | 6 |
| 31. Did you feel tired? | 1 | 2 | 3 | 4 | 5 | 6 |

32. During the **past 4 weeks**, how much of the time has your **physical health or emotional problems** interfered with your social activities (like visiting with friends, relatives, etc.)?

(Circle One Number)

| All of the time | Most of the time | Some of the time | A little bit of the time | None of the time |
| --- | --- | --- | --- | --- |
| 1 | 2 | 3 | 4 | 5 |

How TRUE or FALSE is each of the following statements for you.

(Circle One Number on Each Line)

|  | Definitely True | Mostly True | Don’t Know | Mostly False | Definitely False |
| --- | --- | --- | --- | --- | --- |
| 33. I seem to get ill more easily than other people | 1 | 2 | 3 | 4 | 5 |
| 34. I am as healthy as anybody I know | 1 | 2 | 3 | 4 | 5 |
| 35. I expect my health to get worse | 1 | 2 | 3 | 4 | 5 |
| 36. My health is excellent | 1 | 2 | 3 | 4 | 5 |

**H. Falls**

**Please choose your answer by placing a tick (α) in the box**

| 1a. Have you had any falls including a slip or trip in which you lost your balance and landed on the floor or ground or lower level in the **past 6 months**?  ⬜ 1. Yes  ⬜ 2. No | | | |  |
| --- | --- | --- | --- | --- |
| 1b. **If yes**, how many times have you fallen in **the last 6 months**? | | | |  |
| ⬜ 1 | ⬜ 2 | ⬜ 3 | ⬜ 4 | ⬜ 5 or more |
| 2. Have you experienced a fall **within the last 6 months**, resulting in an injury?   ⬜ 1. Yes  ⬜ 2. No | | | |  |

**I. Feelings**

| Please tell me how much of the time during the last week you felt lonely. | | | |
| --- | --- | --- | --- |
| None or almost none  of the time | Some of the time | Most of the time | All or almost all  of the time |
| 1 ⬜ | 2 ⬜ | 3 ⬜ | 4 ⬜ |

**J. EQ-5D-5L**

Under each heading, please tick the ONE (✔) that best describes

your health TODAY.

| **MOBILITY** |  |
| --- | --- |
| I have no problems in walking about | ⬜ |
| I have slight problems in walking about | ⬜ |
| I have moderate problems in walking about | ⬜ |
| I have severe problems in walking about | ⬜ |
| I am unable to walk about | ⬜ |
|  |  |
| **SELF-CARE** |  |
| I have no problems washing or dressing myself | ⬜ |
| I have slight problems washing or dressing myself | ⬜ |
| I have moderate problems washing or dressing myself | ⬜ |
| I have severe problems washing or dressing myself | ⬜ |
| I am unable to wash or dress myself | ⬜ |
|  |  |
| **USUAL ACTIVITIES** *(e.g. work, study, housework, family or leisure activities)* |  |
| I have no problems doing my usual activities | ⬜ |
| I have slight problems doing my usual activities | ⬜ |
| I have moderate problems doing my usual activities | ⬜ |
| I have severe problems doing my usual activities | ⬜ |
| I am unable to do my usual activities | ⬜ |
|  |  |
| **PAIN / DISCOMFORT** |  |
| I have no pain or discomfort | ⬜ |
| I have slight pain or discomfort | ⬜ |
| I have moderate pain or discomfort | ⬜ |
| I have severe pain or discomfort | ⬜ |
| I have extreme pain or discomfort | ⬜ |
|  |  |
| **ANXIETY / DEPRESSION** |  |
| I am not anxious or depressed | ⬜ |
| I am slightly anxious or depressed | ⬜ |
| I am moderately anxious or depressed | ⬜ |
| I am severely anxious or depressed | ⬜ |
| I am extremely anxious or depressed | ⬜ |

- We would like to know how good or bad


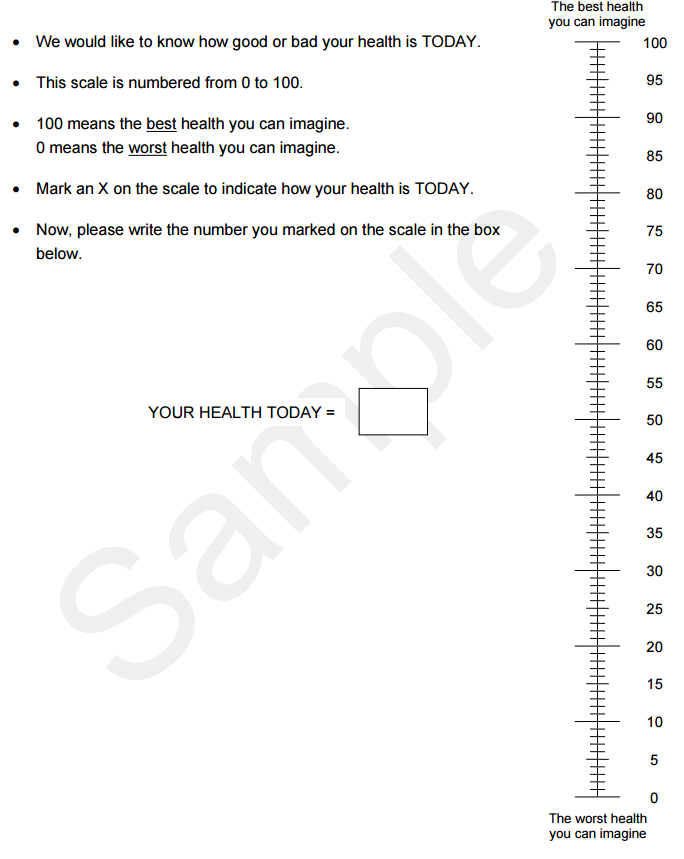


100

95

90

85

80

75

70

65

60

55

50

45

40

35

30

25

20

15

10

5

0

The best health you can imagine

The worst health you can imagine

your health is TODAY.

- This scale is numbered from 0 to 100.
- 100 means the best health you can imagine.

0 means the worst health you can imagine.

- Mark an X on the scale to indicate how your

health is TODAY.

- **Now, please write the number you**

**marked on the scale in the box below.**

YOUR HEALTH TODAY =

**K1. Medical History (baseline only)**

We ask these questions to get a better picture of your overall health and quality of life. Again, all of your information will be kept strictly confidential.

1. How many medications do you currently take regularly?

| 0 ⬜ | 1 ⬜ | 2 ⬜ | 3 ⬜ | 4 ⬜ | 5 or more ⬜ |
| --- | --- | --- | --- | --- | --- |

2. Have you started taking any new medications in the last 6 months?

⬜ No If **NO**, please go to **QUESTION 3**

⬜ Yes If YES, please give details below:

What medications have you started taking?

3. Are you currently being treated for any of the following problems?

CHECK (✔) all that apply.

⬜ a. Rheumatoid arthritis

⬜ b. Diabetes

⬜ c. Chronic Kidney Disease

⬜ d. Osteoporosis

⬜ e. Osteoarthritis

⬜ f. Asthma

⬜ g. COPD

**Cardiovascular Disease**

⬜ h. Coronary Heart Disease

⬜ i. High blood pressure (hypertension)

⬜ j. Atrial Fibrillation

⬜ k. Peripheral Arterial Disease.

⬜ l. Stroke/Transient Ischaemic Attack (TIA-mini stroke)

**Other conditions:**

⬜ m. Epilepsy

⬜ n. Cancer

⬜ o. Learning Disability

4. Have you had any ongoing back pain that limits your mobility?

CHECK (✔) all that apply.

⬜ No If **NO**, please go to **QUESTION 5**

⬜ Yes If **YES**, please give details below:

**If Yes,** could you please indicate the severity of pain experienced during the last week?

| None | Mild | Moderate | Severe | Extreme |
| --- | --- | --- | --- | --- |
| ⬜ | ⬜ | ⬜ | ⬜ | ⬜ |

5. Have you been treated by a health care professional for any of the following?

This question is in 2 parts: first, please think about the LAST 5 YEARS, then think about if you’ve EVER been treated for the condition.

**CHECK all that apply.**

| **Condition** | **Last 5 years** | **Ever** | **Never** |
| --- | --- | --- | --- |
| a. Major depression | ⬜ | ⬜ | ⬜ |
| b. Bipolar disorder | ⬜ | ⬜ | ⬜ |
| c. Schizophrenia | ⬜ | ⬜ | ⬜ |
| d. Psychosis | ⬜ | ⬜ | ⬜ |

**K2. Medical History - Co-interventions and health changes**

**(6, 12 and 24 months only)**

**1.** Since our last meeting, have you attended any physical activity programme or fitness programme outside of this study?

Yes ⬜ No ⬜

⏷

If Yes, could you tell us what this was and how many sessions were involved?

...............................................................................................................................

...............................................................................................................................

..............................................................................................................................

**2.** Were you diagnosed with any kind of non-trivial illness (e.g. cancer, high blood pressure, but not colds or flu) since our last meeting?

Yes ⬜ No ⬜

⏷

If Yes, could you tell us what this was?

...............................................................................................................................

...............................................................................................................................

...............................................................................................................................

**3.** Were you prescribed any new repeat medications since our last meeting?

Yes ⬜ No ⬜

⏷

If Yes, could you tell us what these were?

...............................................................................................................................

...............................................................................................................................

...............................................................................................................................

**4.** Did you have any operations since our last meeting?

Yes ⬜ No ⬜

⏷

If Yes, could you tell us what these were?

...............................................................................................................................

...............................................................................................................................

...............................................................................................................................

**L. Chronic Pain**

**INSTRUCTIONS TO PATIENTS**

The following questions will asked in the following format and you should give your answers by putting an “X” in one of the boxes.

| NOTE: **1. If you put your “X” in the left-hand box, i.e.** | | | | |
| --- | --- | --- | --- | --- |
| None  **☒** | Mild  ⬜ | Moderate  ⬜ | Severe  ⬜ | Extreme  ⬜ |
| Then you are indicating that you have no pain.  **2. If you put your “X” in the right-hand box, i.e.** | | | | |
| None  ⬜ | Mild  ⬜ | Moderate  ⬜ | Severe  ⬜ | Extreme  **☒** |
| Then you are indicating that your pain is extreme. | | | | |

**The following question concern the amount of pain you have experienced in your hip, knee or ankle joints. For each situation please enter the amount of pain experienced during the last week. (Please mark your answers with an )**

| **QUESTION: How much pain do you have?** | | | | | |
| --- | --- | --- | --- | --- | --- |
|  | | | | | |
| **1. Walking on a flat surface.** | | | | |  |
| None  ⬜ | Mild  ⬜ | Moderate  ⬜ | Severe  ⬜ | Extreme  ⬜ |  |
| **2. Going up or down stairs.** | | | | |  |
| None  ⬜ | Mild  ⬜ | Moderate  ⬜ | Severe  ⬜ | Extreme  ⬜ |  |
| **3. At night while in bed.** | | | | |  |
| None  ⬜ | Mild  ⬜ | Moderate  ⬜ | Severe  ⬜ | Extreme  ⬜ |  |
| **4. Sitting or lying.** | | | | |  |
| None  ⬜ | Mild  ⬜ | Moderate  ⬜ | Severe  ⬜ | Extreme  ⬜ |  |
| **5. Standing upright.** | | | | |  |
| None  ⬜ | Mild  ⬜ | Moderate  ⬜ | Severe  ⬜ | Extreme  ⬜ |  |

**M. Resource Use**

**QUESTION 1:**  **HAVE YOU USED ANY OF THE FOLLOWING PRIMARY AND/OR COMMUNITY-BASED SERVICES DURING THE LAST 6 MONTHS?**

*Please tick α* the appropriate box for each type of service

| **Type of service** | **Used this service?** | | **Total number of contacts or consultations in the last 6 months** |
| --- | --- | --- | --- |
| **GP** at surgery/health centre | ⬜ No | ⬜ Yes | ⬜ ⬜ |
| **GP** via telephone | ⬜ No | ⬜ Yes | ⬜ ⬜ |
| **GP** at home | ⬜ No | ⬜ Yes | ⬜ ⬜ |
| **Practice nurse** at surgery/health centre | ⬜ No | ⬜ Yes | ⬜ ⬜ |
| **Practice nurse** via telephone | ⬜ No | ⬜ Yes | ⬜ ⬜ |
| **Practice nurse** at home | ⬜ No | ⬜ Yes | ⬜ ⬜ |
| **Physiotherapist** at surgery/health centre | ⬜ No | ⬜ Yes | ⬜ ⬜ |
| **Physiotherapist** at home | ⬜ No | ⬜ Yes | ⬜ ⬜ |
| **Occupational therapist** at surgery/health centre | ⬜ No | ⬜ Yes | ⬜ ⬜ |
| **Occupational therapist** at home | ⬜ No | ⬜ Yes | ⬜ ⬜ |
| **Nutritionist** | ⬜ No | ⬜ Yes | ⬜ ⬜ |
| **Chiropodist** | ⬜ No | ⬜ Yes | ⬜ ⬜ |
| **Counsellor** | ⬜ No | ⬜ Yes | ⬜ ⬜ |
| **Walk-in-Centre** | ⬜ No | ⬜ Yes | ⬜ ⬜ |
| Other, please specify:  …………………………………………  …………………………………………  ………………………………………… | ⬜ No | ⬜ Yes | ⬜ ⬜ |

**QUESTION 2: HAVE YOU USED ANY DAY CARE SERVICES DURING THE LAST 6 MONTHS?**

*Please tick ✔ one box only*

⬜ No If **NO**, please go to **QUESTION 3**

⬜ Yes If **YES**, please give details below:

| **Type of service (e.g. day centre)** | **How many times did you attend during the last 6 months?** | **How long were you there each time (on average) in minutes?** |
| --- | --- | --- |
| Day care centre | ⬜ ⬜ ⬜ | ⬜ ⬜ |
| Other, please specify  ………………………………… | ⬜ ⬜ ⬜ | ⬜ ⬜ |
| Other, please specify  ………………………………… | ⬜ ⬜ ⬜ | ⬜ ⬜ |

**QUESTION 3: DID YOU STAY IN HOSPITAL OVERNIGHT DURINGTHE LAST 6 MONTHS?**

*Please tick ✔ one box only*

⬜ No If **NO**, please go to **QUESTION 4**

⬜ Yes If **YES**, please give details below:

| **Reason for admission** | **Number of admissions** | **Total number of inpatient nights** |
| --- | --- | --- |
| General medical ward | ⬜ ⬜ ⬜ | ⬜ ⬜ |
| Long-stay ward | ⬜ ⬜ ⬜ | ⬜ ⬜ |
| Intensive care unit | ⬜ ⬜ ⬜ | ⬜ ⬜ |
| Other, please specify  ………………………………… | ⬜ ⬜ ⬜ | ⬜ ⬜ |

**Q4. Did you visit the hospital for an outpatient appointment during the**

**last 6 months?**

*Please tick ✔ one box only*

⬜ No If **NO**, please go to **QUESTION 5**

⬜ Yes If **YES**, please give details below:

Number of outpatient appointments during the last **6 months** ⬜ ⬜ ⬜

**Q5. Did you have any hospital treatment as a day case (not staying**

**overnight) during the last 6 months?**

*Please tick ✔ one box only*

⬜ No If **NO**, please go to **QUESTION 6**

⬜ Yes If **YES**, please give details below:

Number of day case(s) during the last **6 months** ⬜ ⬜ ⬜

**Q6. Did you attend an accident and emergency department (A & E or**

**Casualty) during the last 6 months?**

*Please tick ✔ one box only*

⬜ No If **NO**, please go to **QUESTION 7**

⬜ Yes If **YES**, please give details below:

Number of A&E visits during the last **6 months** ⬜ ⬜ ⬜

**Q7. Have you stayed in a Convalescent or Nursing Home during the last**

**6 months?**

*Please tick ✔ one box only*

⬜ No If **NO**, please go to **QUESTION 8**

⬜ Yes If **YES**, please give details below:

Number of days stayed in a convalescent or nursing home during the last **6 months** ⬜ ⬜ ⬜

**Q8. Have relatives and/or friends helped you with tasks at home which**

**you have had difficulty with or could not do in the last 6 months?**

*Please tick ✔ one box only*

⬜ No If **NO**, please go to **QUESTION 9**

⬜ Yes If **YES**, please give details below:

And you have had help from relatives and/or friends, typically how many hours per week? Hours per week = ⬜ ⬜ ⬜

**Q9: During the last 6 months, have realtives and/or friends stayed off**

**work to help you?**

*Please tick ✔ one box only*

⬜ No If **NO,** Please go to the next section.

⬜ Yes If **YES**, please give details below:

How many days did they take off work in the last **6 months**? Days = ⬜ ⬜ ⬜

**N. Height and Weight**

|  | **1^st^** | **2^nd^** | **3^rd^**  **^(Only conducted if necessary)^** | **Final recorded value** |
| --- | --- | --- | --- | --- |
| **Height** | **cms** | **cms** | **cms** | **cms** |
| **Weight** | **kg** | **kg** | **kg** | **kg** |
| **Shoes worn?** | **Yes/No** |  | | |

**O. Grip Strength result**

| **Attempt 1** |  |
| --- | --- |
| **Attempt 2** |  |
| **Hand used** | **Left/Right** |

**MAT-sf score**

|  |
| --- |

**P. Process evaluation**

**Your attitudes to physical activity and exercise**

The following questions are to help us understand about how people make decisions about how much physical activity and exercise they do. There are no right or wrong answers.

| In the questions below, “**moderate intensity physical activity”** means activities where you work hard enough to make you breathe harder or get your heart beating faster than normal (for example brisk walking, dancing or digging the garden). | | | | | | | | | | | | | | | | | | |  |
| --- | --- | --- | --- | --- | --- | --- | --- | --- | --- | --- | --- | --- | --- | --- | --- | --- | --- | --- | --- |
| *Please circle only one number that you feel best applies to you for each item* | | | | | | | | | | | | | | | | | | |  |
|  | | | | | | | | | | | | | | | | | | |  |
| 1. Doing at least 30 minutes of moderate intensity physical activity on at least 5 days a week **is very important to me**. | | | | | | | | | | | | | | | | | | |  |
| **Not**  **important at all** | | | | | | | | | **Extremely important** | | | | | | | | | |  |
| 0 | | 1 | 2 | 3 | 4 | | 5 | | | | 6 | 7 | | 8 | | 9 | | 10 |  |
| 2**. I am confident in my ability** to do at least 30 minutes of moderate intensity physical activity on at least 5 days a week. | | | | | | | | | | | | | | | | | | |  |
| **Not**  **confident at all** | | | | | | | | | **Extremely confident** | | | | | | | | | |  |
| 0 | | 1 | 2 | 3 | 4 | | 5 | | | | 6 | 7 | | 8 | | 9 | | 10 |  |
| In the questions below, “**Muscle strengthening exercise”** means doing exercises (like squats or repeated lifting of weights) to strengthen your arms, legs and other muscles in your body.  3. Doing muscle-strengthening exercises on at least two days a week is **very important to me**. | | | | | | | | | | | | | | | | | | |  |
| **Strongly**  **Disagree** | | | | | | | | | **Strongly**  **Agree** | | | | | | | | | |  |
| 0 | | 1 | 2 | 3 | 4 | | 5 | | | | 6 | 7 | | 8 | | 9 | | 10 |  |
| 4. **I am confident in my ability** to do two or more sessions of muscle-strengthening exercise each week. | | | | | | | | | | | | | | | | | | |  |
| **Strongly**  **Disagree** | | | | | | | | | **Strongly**  **Agree** | | | | | | | | | |  |
| 0 | | 1 | 2 | 3 | 4 | | 5 | | | | 6 | 7 | | 8 | | 9 | | 10 |  |
|  | | | | | | | |  | | | | | | | | | | |  |
|  | In the following questions: “**moderate intensity physical activity”** means activities where you work hard enough to make you breathe harder or get your heart beating faster than normal (for example brisk walking, dancing or digging the garden) | | | | | | | | | | | | | | | | | | |
|  | *Please circle only one number that you feel best applies to you for each item.* | | | | | | | | | | | | | | | | | | |
|  |  | | | | | **Strongly**  **disagree** | | | | **Dis-**  **agree** | | | **Neither agree or disagree** | | **Agree** | | **Strongly**  **agree** | | |
|  | There are others in my life with whom I can be physically active. | | | | | 1 | | | | 2 | | | 3 | | 4 | | 5 | | |
|  | I feel free to make my own decisions about physical activity. | | | | | 1 | | | | 2 | | | 3 | | 4 | | 5 | | |
|  | There are people in my life who encourage me to be physically active. | | | | | 1 | | | | 2 | | | 3 | | 4 | | 5 | | |
|  | I enjoy being physically active. | | | | | 1 | | | | 2 | | | 3 | | 4 | | 5 | | |
|  | I feel like I am in charge of how often I do physical activity. | | | | | 1 | | | | 2 | | | 3 | | 4 | | 5 | | |
|  | Keeping up my current level of physical activity conflicts with other priorities in my life. | | | | | 1 | | | | 2 | | | 3 | | 4 | | 5 | | |
|  | Keeping up my current level of physical activity over the next year would be a huge struggle. | | | | | 1 | | | | 2 | | | 3 | | 4 | | 5 | | |
|  |  | | | | | | | | | | | | | | | | | | |
|  | In the following questions: **“Muscle strengthening exercise”** means doing exercises (like squats or repeated lifting of weights) to strengthen your arms, legs and other muscles in your body | | | | | | | | | | | | | | | | | | |
|  | *Please circle only one number that you feel best applies to you for each item.*  Not applicable (I have not done any muscle strengthening exercises) | | | | | | | | | | | | | | | | | | |
|  |  | | | | | **Strongly**  **disagree** | | | | **Dis-**  **agree** | | | **Neither agree or disagree** | | **Agree** | | **Strongly**  **agree** | | |
|  | There are others in my life with whom I can do muscle-strengthening exercises. | | | | | 1 | | | | 2 | | | 3 | | 4 | | 5 | | |
|  | I feel free to make my own decisions about muscle-strengthening exercises. | | | | | 1 | | | | 2 | | | 3 | | 4 | | 5 | | |
|  | There are people in my life who encourage me to do muscle-strengthening exercises. | | | | | 1 | | | | 2 | | | 3 | | 4 | | 5 | | |
|  | I enjoy doing muscle-strengthening exercises. | | | | | 1 | | | | 2 | | | 3 | | 4 | | 5 | | |
|  | I feel like I am in charge of how often I do muscle-strengthening exercises. | | | | | 1 | | | | 2 | | | 3 | | 4 | | 5 | | |
|  | Keeping up my current level of muscle-strengthening exercise conflicts with other priorities in my life. | | | | | 1 | | | | 2 | | | 3 | | 4 | | 5 | | |
|  | Keeping up my current level of muscle strengthening exercise over the next year would be a huge struggle. | | | | | 1 | | | | 2 | | | 3 | | 4 | | 5 | | |

**Q. Feedback on the REACT programme**

**(6 and 12 months *intervention* group only)**

| *Please circle only one number that you feel best applies to you* | | | | | |
| --- | --- | --- | --- | --- | --- |
|  | **Strongly**  **disagree** | **Dis-**  **agree** | **Neither agree or disagree** | **Agree** | **Strongly**  **Agree** |
| I identified with our group leaders. | 1 | 2 | 3 | 4 | 5 |
| I felt understood and listened to by our group leaders. | 1 | 2 | 3 | 4 | 5 |
| The group leaders encouraged us to ask questions. | 1 | 2 | 3 | 4 | 5 |
| The group leaders tried to understand how we see things before suggesting new ways to do things. | 1 | 2 | 3 | 4 | 5 |
| Other members in the REACT group meetings really try to help me. | 1 | 2 | 3 | 4 | 5 |
| I identify with other members in my REACT group. | 1 | 2 | 3 | 4 | 5 |
| I feel strong ties with other members in my REACT group. | 1 | 2 | 3 | 4 | 5 |

On a scale from 1-10, how much did you enjoy the REACT programme?

*Please circle the number that best* *describes how much you enjoyed the REACT programme. Your exercise instructor will not see your answer, so please answer honestly.*

| **Not at all** | | | | | **Extremely** | | | | | |
| --- | --- | --- | --- | --- | --- | --- | --- | --- | --- | --- |
| 1 | 2 | 3 | 4 | 5 | | 6 | 7 | 8 | 9 | 10 |

| What did you like the most? (Please comment below) |
| --- |
|  |

| What did you like the least? (Please comment below) |
| --- |
|  |

On a scale from 0 to 10, with 0 being the worst score and 10 being the best, how likely is it that you would recommend the REACT programme to a family member or friend?

*Please circle only one number that you feel best applies to you.*

| **Extremely**  ***Un*likely** | | | | | **Extremely**  **likely** | | | | | |
| --- | --- | --- | --- | --- | --- | --- | --- | --- | --- | --- |
| 1 | 2 | 3 | 4 | 5 | | 6 | 7 | 8 | 9 | 10 |

**R. Muscle-strengthening exercise questionnaire**

**(0, 6, 12, 24 months, both groups)**

The UK Department of Health recommends that older adults should do **muscle-strengthening exercises** on at least two days a week to improve their quality of life and reduce health risks. This means doing exercises (like squats, moving heavy loads like groceries, or repeated lifting of weights) to strengthen your arms, legs and other muscles in your body.

*Please circle only one number that you feel best applies to you for each item.*

|  | **Strongly**  **disagree** | | **Dis-**  **Agree** | **Neither agree or disagree** | **Agree** | **Strongly**  **agree** | |
| --- | --- | --- | --- | --- | --- | --- | --- |
| I normally do less muscle-strengthening exercise than recommended above. | | 0 | 1 | 2 | 3 | 4 |  |
| I don’t have time to do muscle-strengthening exercises. | | 0 | 1 | 2 | 3 | 4 |  |
| I sometimes do the recommended amount of muscle-strengthening exercise. | | 0 | 1 | 2 | 3 | 4 |  |

Please answer the following in relation to your own recent experiences of doing any muscle-strengthening exercises (or not) over the last few months. If you have not done any such exercise, feel free to “disagree” with the statements.

**“Muscle strengthening exercise”** means doing exercises (like squats, moving heavy loads like groceries, or repeated lifting of weights) to strengthen your arms, legs and other muscles in your body.

| *Please circle only one number that you feel best applies to you for each item*  Not applicable (I have not done any muscle strengthening exercises) | | | | | |
| --- | --- | --- | --- | --- | --- |
|  | **Strongly**  **disagree** | **Dis-**  **agree** | **Neither agree or disagree** | **Agree** | **Strongly**  **Agree** |
| **Based on my recent experiences …** | |  |  |  |  |
| The muscle-strengthening exercises that I have done have helped me to feel fit and healthy. | 1 | 2 | 3 | 4 | 5 |
| Doing muscle-strengthening exercises has increased my overall energy levels. | 1 | 2 | 3 | 4 | 5 |
| Doing muscle-strengthening exercises has improved my mood. | 1 | 2 | 3 | 4 | 5 |
| Doing muscle-strengthening exercises has had benefits for my social life. | 1 | 2 | 3 | 4 | 5 |

**Thank you!**

**That’s it – thank you very much for completing the measurement session**! We hope that you have enjoyed thinking about some of these ideas.

**If the participant has any questions you cannot answer**, please ask them to contact your local PI, Janet Withall (j.withall@bath.ac.uk) or Afroditi Stathi (A. Stathi@bath.ac.uk) or call 01225 385449.
